# Outer membrane and phospholipid composition of the target membrane affect the antimicrobial potential of first- and second-generation lipophosphonoxins

**DOI:** 10.1038/s41598-021-89883-0

**Published:** 2021-05-17

**Authors:** Klára Látrová, Noemi Havlová, Renata Večeřová, Dominik Pinkas, Kateřina Bogdanová, Milan Kolář, Radovan Fišer, Ivo Konopásek, Duy Dinh Do Pham, Dominik Rejman, Gabriela Mikušová

**Affiliations:** 1 Department of Genetics and Microbiology, Faculty of Science, Charles University, Viničná 5, 128 00 Prague 2, Czech Republic; 2 Department of Microbiology, Faculty of Medicine and Dentistry, Palacký University Olomouc, Hněvotínská 3, 775 15 Olomouc, Czech Republic; 3 Institute of Organic Chemistry and Biochemistry, Czech Academy of Sciences v.v.i., Flemingovo nám. 2, 166 10 Prague 6, Czech Republic

**Keywords:** Ion channels, Antibiotics, Single-molecule biophysics, Drug discovery, Drug discovery and development

## Abstract

Lipophosphonoxins (LPPOs) are small modular synthetic antibacterial compounds that target the cytoplasmic membrane. First-generation LPPOs (LPPO I) exhibit an antimicrobial activity against Gram-positive bacteria; however they do not exhibit any activity against Gram-negatives. Second-generation LPPOs (LPPO II) also exhibit broadened activity against Gram-negatives. We investigated the reasons behind this different susceptibility of bacteria to the two generations of LPPOs using model membranes and the living model bacteria *Bacillus subtilis* and *Escherichia coli*. We show that both generations of LPPOs form oligomeric conductive pores and permeabilize the bacterial membrane of sensitive cells. LPPO activity is not affected by the value of the target membrane potential, and thus they are also active against persister cells. The insensitivity of Gram-negative bacteria to LPPO I is probably caused by the barrier function of the outer membrane with LPS. LPPO I is almost incapable of overcoming the outer membrane in living cells, and the presence of LPS in liposomes substantially reduces their activity. Further, the antimicrobial activity of LPPO is also influenced by the phospholipid composition of the target membrane. A higher proportion of phospholipids with neutral charge such as phosphatidylethanolamine or phosphatidylcholine reduces the LPPO permeabilizing potential.

## Introduction

The accelerating occurrence of bacterial resistance to current antibiotics poses a constant threat, because it is widely associated with the failure of antibiotic treatment. It was reported that in the USA alone, over 2 million illnesses per year are caused by multi-drug-resistant bacteria and associated annual costs for treating such infections range from $20–$35 billion^1^. This situation has prompted the search for new antimicrobial agents. In this respect, an enormous amount of effort has been focused on antimicrobial peptides (AMPs) and lipopeptides. On the one hand, AMPs offer a number of advantages such as broad-spectrum antimicrobial activities and diverse modes of action such as rapid disruption of microbial cell membranes and thus a lower risk of the development of resistance. On the other hand, the usage of AMPs is often limited to topical applications because of systemic toxicities, low solubility, low in vivo stability, limited tissue biodistribution, and high cost for large-scale manufacturing. Out of more than 3000 AMPs that have been discovered, there are several AMPs with antibacterial activity (e.g. Gramicidin, daptomycin, colistin, vancomycin, oritavancin, dalvabacin, telavancin, bacitracin, polymyxin B, teicoplanin, streptogramin, and tyrothricin) that have been approved by the US Food and Drug Administration^2,3^.

The discovery that the pharmacophore of AMPs is smaller than anticipated and knowledge of the structural features of active AMPs led to the development of small-molecular analogues of AMPs, synthetic antimicrobial peptidomimetics (SAPs), i.e. synthetic mimics of AMPs. AMPs typically adopt several structural features such as facially amphiphilic topology with hydrophilic and hydrophobic side chains segregating to opposing regions, which is essential for interaction, insertion into membrane and subsequent membrane disruption^4^. Another feature common to all AMPs is their positive charge that provides an electrostatic interaction with the negatively charged membrane surface^5^. It is generally believed that their physicochemical properties rather than primary amino acid sequence are responsible for the antimicrobial activity of AMPs.

SAPs seem to be promising because of their relative simplicity and thus easy and cost-effective production. Further, chemical synthesis also offers the use of nonproteinaceous building blocks and the possibility of obtaining SAPs in large amounts with improved properties with respect to their antimicrobial potency, selectivity, stability and biodistribution. However, the challenge of their design is to ensure selectivity while maintaining activity against bacterial pathogens. Whether the target site of an AMP (or SAP) is the cytoplasmic membrane or it has some other intracellular targets, AMP has to interact with the cell surface and reach or at least overcome the membrane. The amphipathic nature of AMPs ensures the primary interaction^6^. Positively charged hydrophilic moiety of an AMP is electrostatically attracted to the negatively charged bacterial surface components such as lipopolysaccharides (LPS), teichoic and teichouronic acids (in Gram-negative and Gram-positive bacteria, respectively) or negatively charged phospholipid headgroups. This interaction occurs less favorably in eukaryotic cells as they contain neutral lipids in the outer leaflet of their plasma membrane. The hydrophobic moiety of an AMP facilitates the interaction with the hydrophobic milieu of the membrane and inserts to it. On the other hand, increased hydrophobicity results in the loss of selectivity^7^. Thus, the general strategy is to properly balance the nature and number of appropriate cationic groups, charge distribution, hydrophobicity, and amphipathicity^8^ as these characteristics determine activity in bacteria as well as selectivity^9^. The relative influence with which each of these parameters contribute to the potency and selectivity differs among different scaffolds.

The search for new antimicrobial compounds needs more studies of AMPs or SAPs which are active against Gram-negative pathogens (e.g. *Acinetobacter baumannii*, *Pseudomonas aeruginosa*, *Klebsiella pneumoniae*) that cause serious infections. Gram-negative bacteria are generally less susceptible towards antimicrobial agents because of their outer membrane, which itself acts as a permeability barrier. The presence of LPS represents a barrier to most hydrophobic as well as large hydrophilic molecules. LPS creates two potential barriers—the hydrophilic one provided by the densely packed oligosaccharide core^10^, and the other hydrophobic one provided by the hydrocarbon chain region of the lipid A^11^. Thus, overcoming the LPS barrier is challenging. A considerable amount of research has been done on polymyxin B derivatives in order to overcome the limitations of its use and enhance efficacy. Some derivatives (such as polymyxin B nonapeptide) might also serve to facilitate passage of other agents across the outer membrane of Gram-negative bacteria^12,13^. Polymyxin structure (cyclic heptapeptide core linked to a linear tripeptide with an N-terminal fatty acyl moiety) and the knowledge that the lipid chain is responsible for its activity^14^ has inspired research on N-lipidated peptide dimers, which are also effective antibacterial agents against Gram-negative pathogens^15^, or lipidation of AMPs in general^16^.

**Figure 1:**
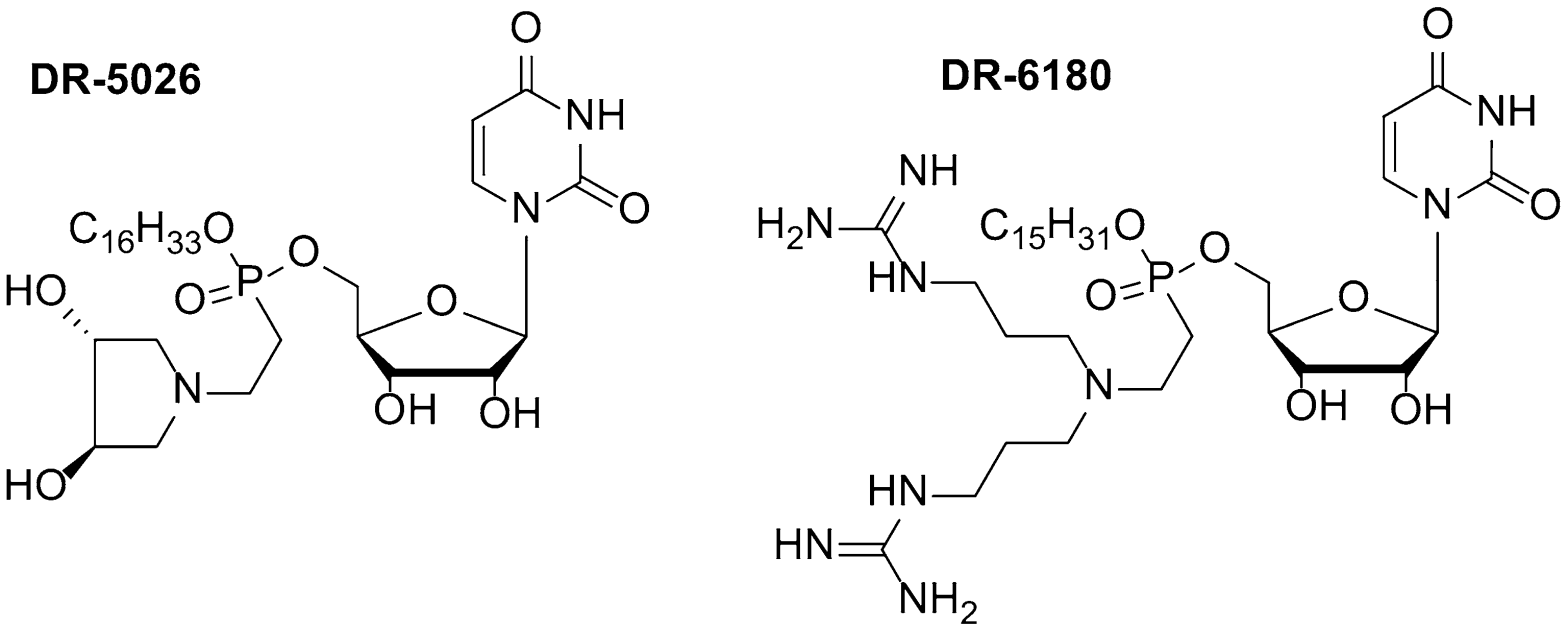
Selected examples of first- (DR-5026) and second-generation (DR-6180) LPPOs used in this study.

Recently, we reported^17–19^ the synthesis of novel compounds termed lipophosphonoxins (LPPOs), a type of modular nonproteinaceous SAPs. LPPOs exhibit significant antibacterial activity against a wide range of bacteria, including multi-resistant strains while they do not exert any adverse effect on eukaryotic cells at bactericidal concentrations. Using living cells we have shown that LPPOs act through the permeabilization of the bacterial membrane, which finally leads in the loss of its barrier function and cell death. Using planar lipid membranes we directly proved LPPOs to be pore-forming agents. In the first generation of LPPOs (LPPO I)^17,19^, the polar module is represented by a hydrophilic moiety with a small positive charge, the hydrophobic module is a linear alkyl chain (C14-16), and the auxiliary module is the nucleoside uridine (Fig. 1). LPPO I only possesses activity against Gram-positive bacteria such as methicillin-resistant *Staphylococcus aureus* or vancomycin-resistant *Enterococccus faecium*. In the LPPO II generation^18^, we increased the positive charge of the polar module, while hydrophobic and auxiliary modules remained unchanged. This structural modification resulted in a broad-spectrum antimicrobial activity against both Gram-positive and Gram-negative bacteria, including clinically significant pathogens such as *Escherichia coli*, *Pseudomonas aeruginosa* or *Salmonella enterica*.

In this work, we conducted a study in order to establish the reasons behind the different activity of LPPOs I and LPPOs II against Gram-positive and Gram-negative bacterial cells and to see why LPPOs II have higher activity against Gram-positive cells. We selected two candidate compounds from each LPPO generation and two model bacteria (Table 1)—the Gram-positive bacterium *Bacillus subtilis* and Gram-negative bacterium *Escherichia coli*. We compared LPPO I and II pore-forming activity on model membranes as well as in living cells, and we show here that both the phospholipid composition and presence of outer membrane affect LPPO antimicrobial activity.

**Table 1:** Antimicrobial and hemolytic activity of selected first- and second-generation LPPOs.

LPPO	MIC (µg/ml)		HC_50_ (µg/ml)
	*Bacillus subtilis*	*Escherichia coli*	
I—DR-5026	3.125	> 200	25.0
II—DR-6180	0.390	0.780	20.0^a^

Antimicrobial and hemolytic activity are expressed as minimum inhibitory concentration (MIC) and the concentration causing lysis of 50% of red blood cells (HC_50_), respectively.

^a^Previously 16 µg/ml^18^, volunteer blood donor changed.

## Results

### Pore formation by LPPO is concentration-dependent

**Figure 2:**
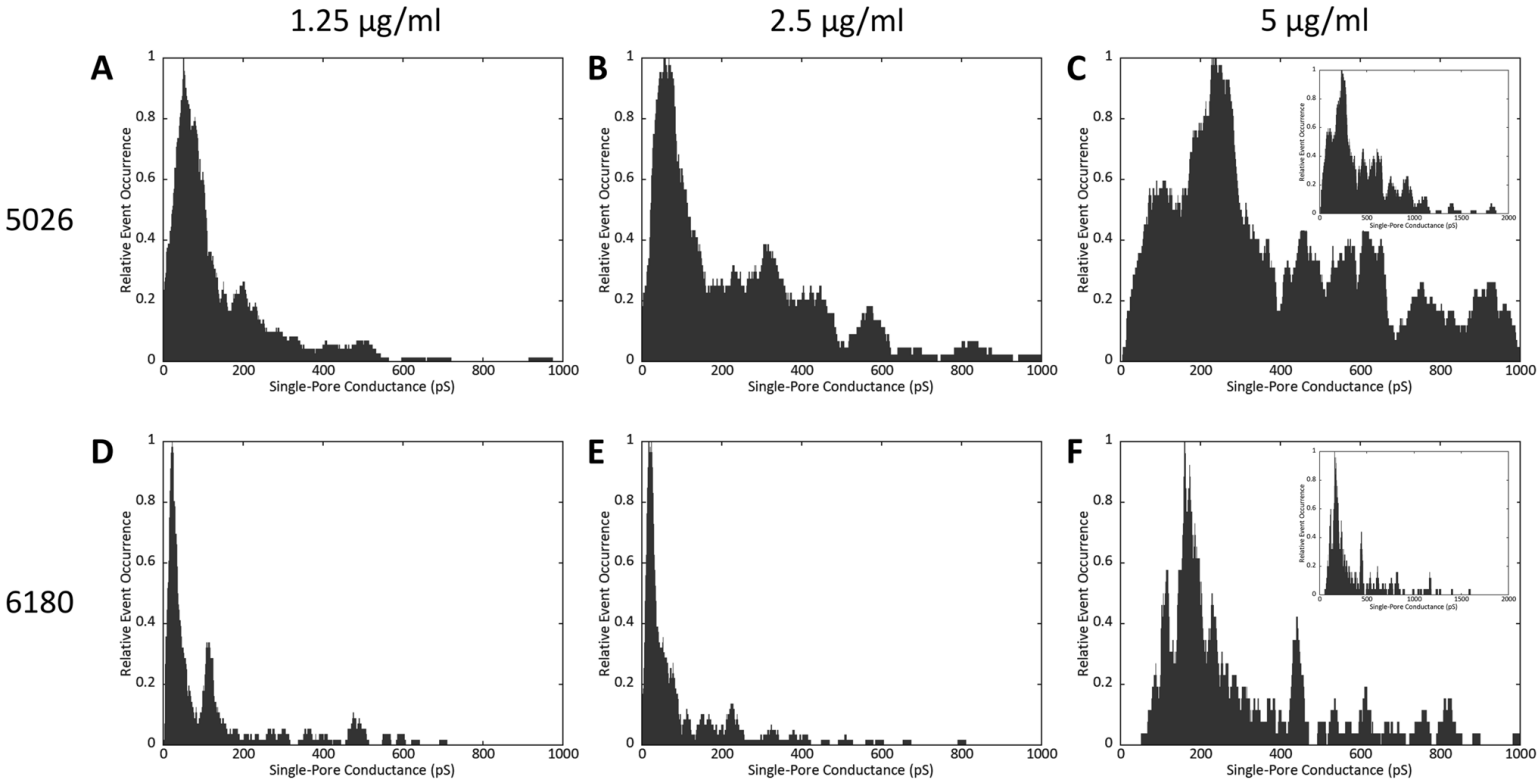
Concentration dependence of LPPO I and II pore formation. (**A**–**C**) LPPO I (DR-5026) single-pore openings, (**D**–**F**) LPPO II (DR-6180) single-pore openings in diphytanoylphosphatidylglycerol membranes, 1 M KCl, 10 mM Tris, pH 7.4, 50 mV. The histograms were created from pore openings using kernel density estimation (rectangular kernel with 30 pS width). The typical current recordings are shown in Supplementary Fig. S1.

Our previous studies on LPPO I and II showed that the mode of action of both LPPO generations is pore formation^17,18^. Currently, we wanted to compare the pore-forming activity of LPPO I (DR-5026) and II (DR-6180) with respect to their concentration dependence. Using conductance measurements on planar lipid membranes composed of diphytanoylphosphatidylglycerol we show (Fig. 2) that both LPPO I and II form a continuum of different pores—there appeared pores which were well resolved but there were also ones with high current noise and with fast dynamics (Supplementary Fig. S1, the raw recorded data are available online—for details see “Methods”). The distribution of LPPO I and II pore conductances was very broad and ranged from tens of pS up to nS in 1 M KCl. At 5 µg/ml (Fig. 2C,F) the most frequent pore conductance is ~ 240 and 160 pS for LPPO I and II, respectively; however, pores up to 2 nS were also seen. It is apparent that LPPO activity is substantially concentration-dependent—with DR-5026, lowering the concentration from 5 to 2.5 µg/ml (Fig. 2B,C) results in lowering the most frequent pore conductance fourfold to ~ 60 pS and the disappearance of pores larger than 1000 pS. Further lowering DR-5026 concentration to 1.25 µg/ml did not change the value of the most frequent pore conductance, but led to a subsequent diminishing of pores larger than 600pS (Fig. 2A). For DR-6180, the tendencies are even more pronounced—lowering the concentration from 5 to 2.5 µg/ml resulted in lowering the most frequent conductance state eightfold to ~ 20 pS (Fig. 2E,F). Using DR-6180 at a concentration of 1.25 µg/ml (Fig. 2D) neither changed the most frequent conductance state nor the overall character of the histogram. The only exception was the appearance of a minor peak at ~ 115 pS, which is also visible at 5 µg/ml. The existence of several discrete conductance states in the histogram implies that LPPOs form oligomer channels with various stoichiometries. The constant pore conductance at lower LPPO concentrations suggests that there may be a smallest conductance state of a defined number of monomers.

### LPPO I induces PI uptake in *B. subtilis* but not in *E. coli*

**Figure 3:**
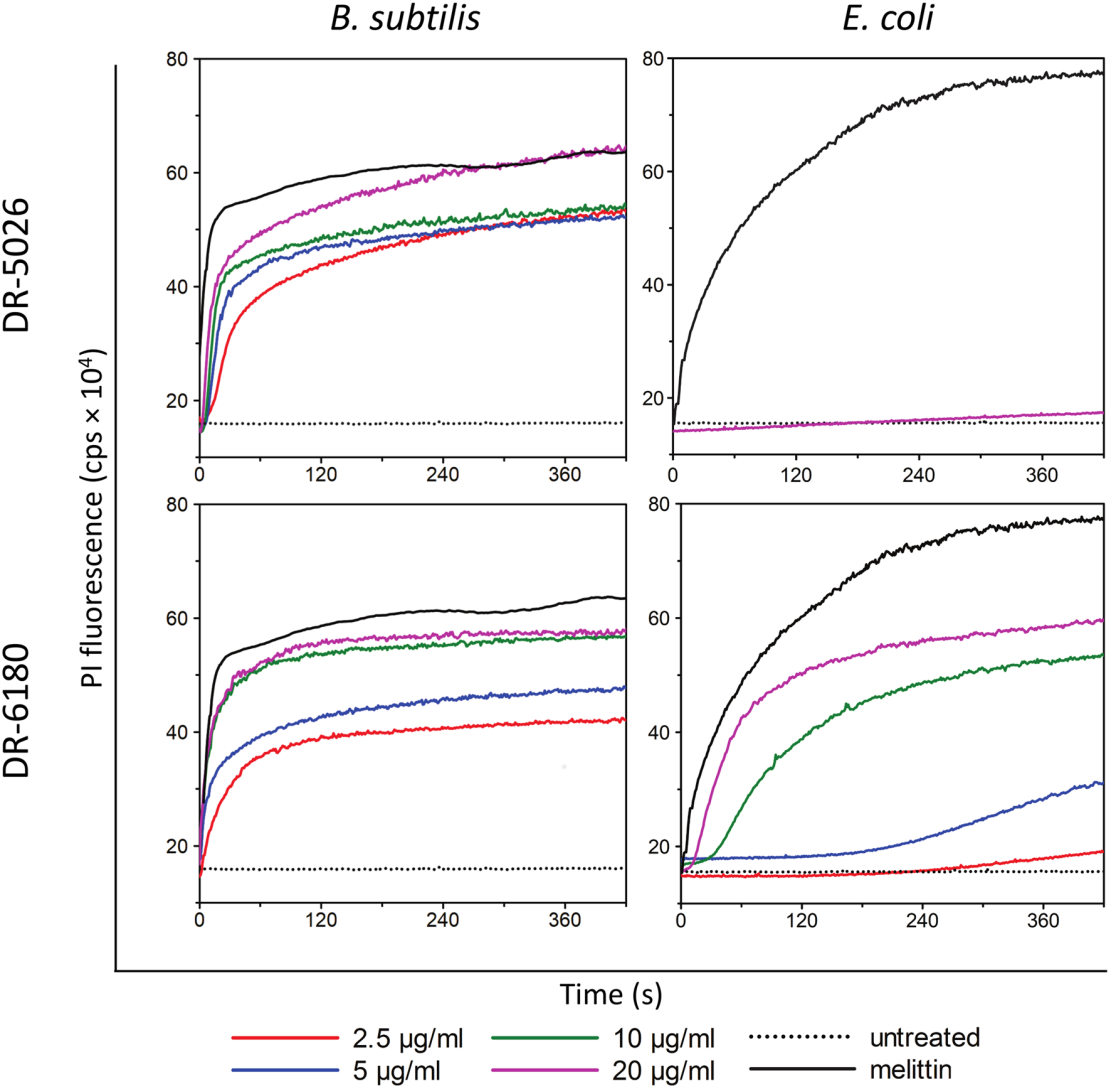
Permeabilization of *B. subtilis* and *E. coli* membrane induced by LPPO. Concentration dependence of LPPO I- (DR-5026) and LPPO II- (DR-6180) induced membrane permeabilization was measured as the increase in fluorescence intensity of the probe propidium iodide. The black dotted line (“untreated”) shows the fluorescence of PI in the suspension of cells without LPPO addition. Melittin at a concentration of 10 µM served as a positive control. Representative kinetics from at least three independent experiments performed in duplicate are shown. With the exception of *B. subtilis* exposed to 2.5 µg/ml DR-5026 the kinetics of PI entry correlated with growth inhibition of the tested bacteria in the presence of LPPOs (Supplementary Fig. S2).

We measured the concentration dependence of LPPO permeabilizing activity in living *B. subtilis* and *E. coli* cells using the membrane-impermeable dye propidium iodide (PI). PI does neither penetrate the outer nor the inner membrane and only enters the cells after membrane damage. Upon entry PI binds to nucleic acids which increases its fluorescence. We tested the permeabilizing activity of the LPPOs in the concentration range of 2.5–20 µg/ml (Fig. 3). All of the LPPO concentrations used added to the *B.* *subtilis* suspension induced an immediate rise in PI fluorescence, suggesting that the dye entered the cells after their permeabilization. The observed effect of LPPO did not change much with respect to the LPPO concentration used—lowering the concentration from 20 to 2.5 µg/ml only reduced the maximum PI fluorescence induced by LPPO by 30%. As expected, when using LPPO I against *E. coli*, we confirmed the data of the minimum inhibitory concentration, which exhibited inactivity against Gram-negative bacteria (Table 1). In the experiment with *E. coli*, we did not observe any rise in fluorescence intensity, even using the highest LPPO I concentration. At the same time, LPPO II permeabilized the *E.* *coli* inner membrane. In contrast to *B. subtilis*, the onset of the PI fluorescence intensity increase was delayed, and the overall kinetics were slower. Both of these effects were concentration-dependent—lowering the LPPO II concentration prolonged the initial delay and slowed the rise in PI fluorescence intensity. The maximum permeabilizing activity was also affected to a great extent by concentration. Concentrations of 20 and 10 µg/ml had roughly the same permeabilizing effect; however, 5 µg/ml resulted in a much more pronounced decrease in the effect.

### LPPO I is capable of inducing partial dissipation of membrane potential of *E. coli*

The potency of LPPOs to permeabilize the cytoplasmic membrane apparently affects membrane potential. Thus, we further wanted to know how LPPO I and II differ in their effectivity to disrupt the membrane potential of living *B. subtilis* and *E. coli* cells. We also wanted to test whether LPPO I DR-5026, even though it is not lethal to the cells of the Gram-negative bacterium *E. coli* (according to MICs), is able to at least partially depolarize the membrane. We used membrane-bound fluorescence probe DiSC_3_(5), which has a high affinity for hyperpolarized membranes. After binding to the membrane, its fluorescence is quenched. The addition of a pore-forming substance leads to depolarization of the membrane, which releases the probe from the membrane and the intensity of fluorescence rises.

**Figure 4:**
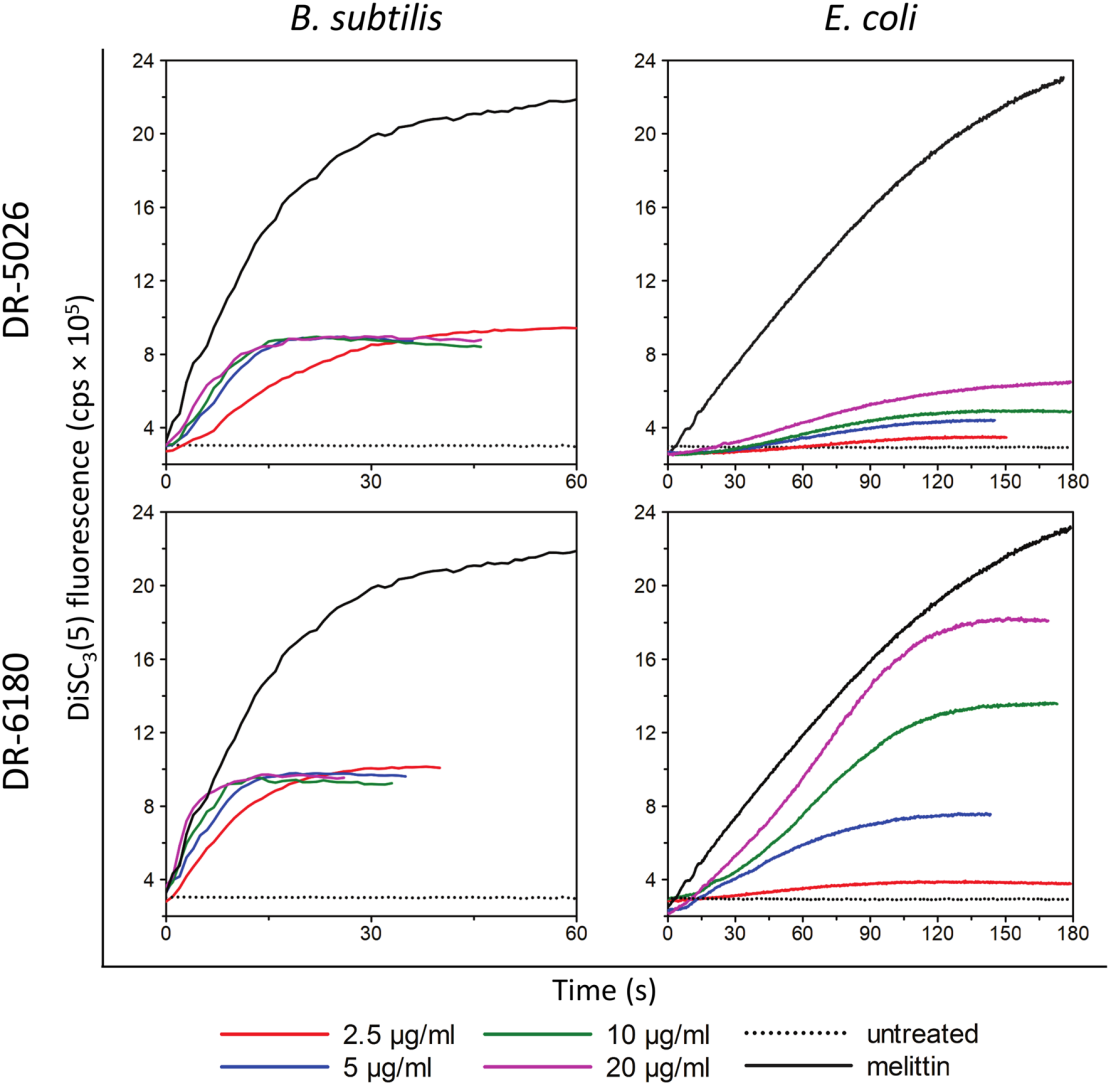
Dissipation of membrane potential of *B. subtilis* and *E. coli* induced by LPPO. Concentration dependence of LPPO I- (DR-5026) and LPPO II- (DR-6180) induced membrane permeabilization was measured as the increase in fluorescence intensity of the probe DiSC_3_(5), which corresponds to the disruption of physiological membrane potential. The black dotted line (“untreated”) shows fluorescence of DiSC_3_(5) in the suspension of cells without LPPO addition. Melittin at a concentration of 10 µM served as a positive control. Representative kinetics from at least three independent experiments performed in duplicate are shown.

In line with the observed permeabilizing activity that was followed via PI, Fig. 4 shows that at any concentration used, both LPPOs are able to disrupt the membrane potential of *B. subtilis*. In this bacterium the depolarization monitored by DiSC_3_(5) fluorescence was, similarly to the kinetics of PI entry, again very rapid, as the maximum fluorescence was reached within 10–20 s. We did not observe any differences between the kinetics with LPPO I and II, and also almost no concentration dependency with regard to both the time-course and the maximum of fluorescence intensity. When using LPPO II against *E. coli*, we observed that the onset of the DiSC_3_(5) fluorescence increase appeared after a delay taking several seconds, and the overall rate of depolarization was much slower than that of *B. subtilis*. At odds with *B. subtilis*, where the time required to reach half of the fluorescence maximum was only on the order of seconds, in *E. coli* it was more than one minute when using 20 µg/ml LPPO I or LPPO II. LPPO II depolarized the *E. coli* cytoplasmic membrane in a concentration-dependent manner—increasing the LPPO II concentration from 2.5 up to 10 µg/ml led to a linear increase in the fluorescence intensity maxima. Note that there is no detectable artificial increase of intensity after mixing of LPPO and DiSC_3_(5) alone in the buffer (Supplementary Fig. S3). Enhancing the concentration to 20 µg/ml did not result in doubling the maximum of the 10 µg/ml concentration. Surprisingly, even when we added LPPO I to the *E. coli* suspension, we detected a slight increase in DiSC_3_(5) fluorescence which was concentration-dependent. Interestingly, when using the highest 20 µg/ml LPPO I concentration, the fluorescence maximum reached was comparable with 5 µg/ml of LPPO II. Thus LPPO I was able to partially disrupt the membrane potential of *E. coli*. This means that some LPPO I molecules inserted into the cytoplasmic membrane, where they formed small pores enabling the flux of ions. On the other hand, the concentration was not high enough to form bigger oligomers that allowed larger molecules such as PI to pass through (Fig. 3).

### Membrane potential of the target cell does not affect the permeabilizing activity of LPPO I nor LPPO II

**Figure 5:**
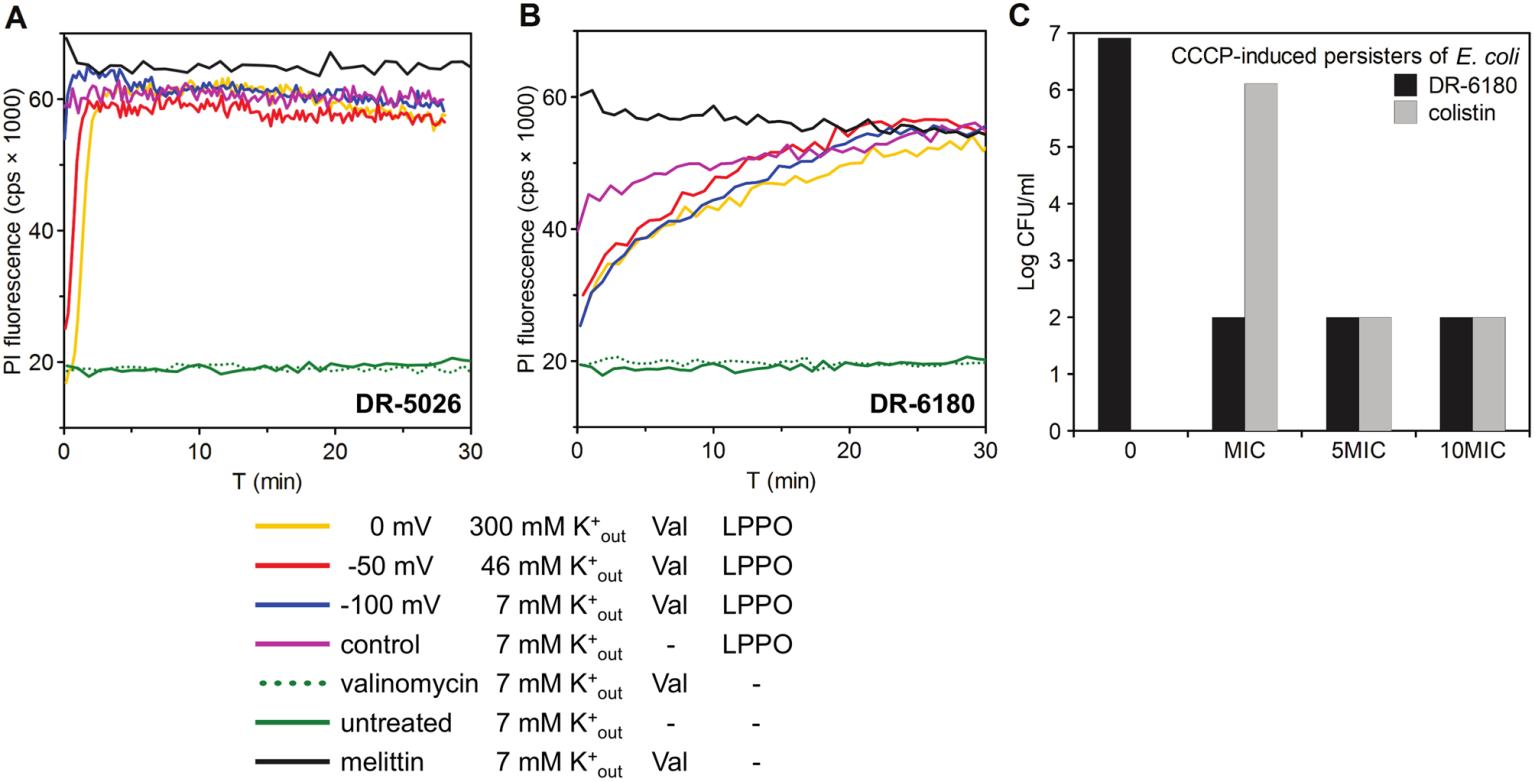
Effect of membrane potential of target cells on LPPO-induced permeabilization. (**A**,**B**) Membrane permeabilization induced by (**A**) LPPO I (DR-5026) and (**B**) LPPO II (DR-6180) at the concentration of 5 µg/ml was measured in *B. subtilis* via the entry of propidium iodide. The cells were resuspended in buffers with different K^+^_out_ concentrations (indicated in the graph legend) and the membrane potential was adjusted to the desired value of ΔΨ (in millivolts) by the addition of 4 µM valinomycin (for details, see “Materials and methods”). Untreated and valinomycin (Val) control kinetics for a buffer with a 7 mM K^+^_out_ concentration are shown. Representative results from three independent experiments performed in duplicate are shown. None of the values of adjusted membrane potential affected the activity of the positive control of melittin (10 µM). For clarity, only the kinetics for ΔΨ − 100 mV (7 mM K^+^_out_ buffer with 4 µM valinomycin) are presented. (**C**) Activity of LPPO II DR-6180 and colistin against CCCP-induced persisters of *E. coli*. Antimicrobial activity was evaluated by CFU/ml counting after three hours of incubation of persisters with the tested compound at concentrations corresponding to MIC (minimal inhibitory concentration), 5 × MIC and 10 × MIC. The value of 10^2^ CFU/ml was used as the detection limit.

We next decided to test whether the permeabilizing activity of LPPO I and II is influenced by the value of membrane potential in living *B. subtilis* cells with adjusted membrane potential. We prepared three suspensions of *B. subtilis* cells in buffers differing in their KCl (K^+^_out_) concentration. We set the membrane potential using valinomycin (for details see “Materials and methods”) to values of − 100, − 50 and 0 mV, and afterwards we added LPPO. We compared the kinetics of LPPO-induced PI entry into the cells treated with valinomycin with the intact (valinomycin-untreated) ones which had the physiological value of membrane potential. Figure 5A shows that the permeabilizing activity of LPPO I (DR-5026) was independent of the value of membrane potential. When using LPPO II (DR-6180, Fig. 5B) the kinetics of permeabilization of cells with adjusted membrane potential was clearly slower; however, the intensity reached the same maximum values as in the intact cells.

We further verified the finding that LPPO activity is not affected by the value of membrane potential by assessing the activity of LPPO II against a persister culture of *E. coli* (Fig. 5C). LPPO II at its MIC eradicated persisters of *E. coli* below the limit of detection (10^2^ CFU/ml). At the same time, colistin reduced bacteria persister-enriched suspension below the detection limit at a concentration 5 × MIC.

### LPPO I does not compromise the outer membrane of *E. coli*

**Figure 6:**
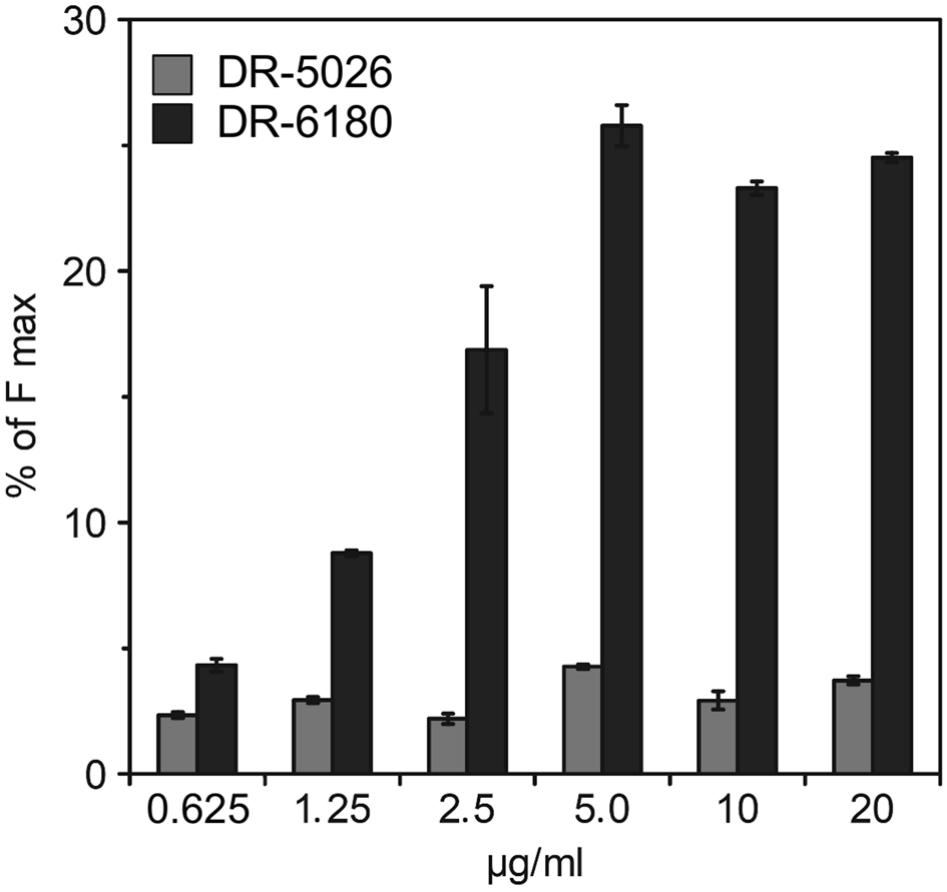
*Escherichia coli* outer membrane permeability induced by LPPO. The integrity of the outer membrane was assessed by measuring the increase in fluorescence intensity due to NPN uptake by cells with a compromised outer membrane induced by LPPO. The data represent the percentage of fluorescence intensity from the entire kinetics plot after reaching the plateau relative to the maximum induced by the addition of 100 µM polymyxin B.

We investigated the reasons why LPPO I are ineffective against Gram-negative cells of *E. coli*. We hypothesized that LPPO I are unable to disintegrate the outer membrane, which is the first hurdle that must be overcome to reach the target site—the cytoplasmic membrane. To follow the changes in the integrity of the outer membrane, we used the probe NPN, the fluorescence of which increases when the outer membrane is compromised. The data in Fig. 6 (and Supplementary Fig. S5) clearly show that LPPO II DR-6180 is capable of compromising the integrity of the outer membrane. The effect reaches its maximum at a concentration of 5 µg/ml. Using this DR-6180 concentration led to a level of disintegration of the outer membrane that was 26% of that induced by polymyxin B. Increasing the concentration up to 10 and 20 µg/ml did not enhance the efficiency of outer membrane permeabilization. In contrast, LPPO I DR-5026 was almost incapable of compromising the integrity of the outer membrane—irrespective of the concentration used, the efficiency of outer membrane permeabilization compared to that of polymyxin B was at most 4%.

### Phospholipid composition of the target membrane affects LPPO activity

We next tested another hypothesis, that besides the presence of the outer membrane, the phospholipid composition of the target inner membrane also affects LPPO activity. Whereas in *E. coli* phosphatidylethanolamine represents the major phospholipid of the inner membrane^20^, in case of *B. subtilis* it is phosphatidylglycerol^21^. We prepared carboxyfluorescein-loaded liposomes composed of phospholipid mixtures in a 2:1 (w/w) ratio which resembled the proportion of the two major phospholipids in the *B.* *subtilis* and *E. coli* cytoplasmic membrane. The mixtures contained phosphatidylglycerol/phosphatidylethanolamine (PG/PE) or phosphatidylethanolamine/phosphatidylglycerol (PE/PG) in order to represent *B. subtilis* and *E.* *coli* phospholipid composition of the inner membrane, respectively. The third membrane system was composed of neutral phosphatidylcholine/phosphatidylethanolamine (PC/PE) and represented characteristic phospholipids from the outer leaflet of the eukaryotic plasma membrane.

**Figure 7:**
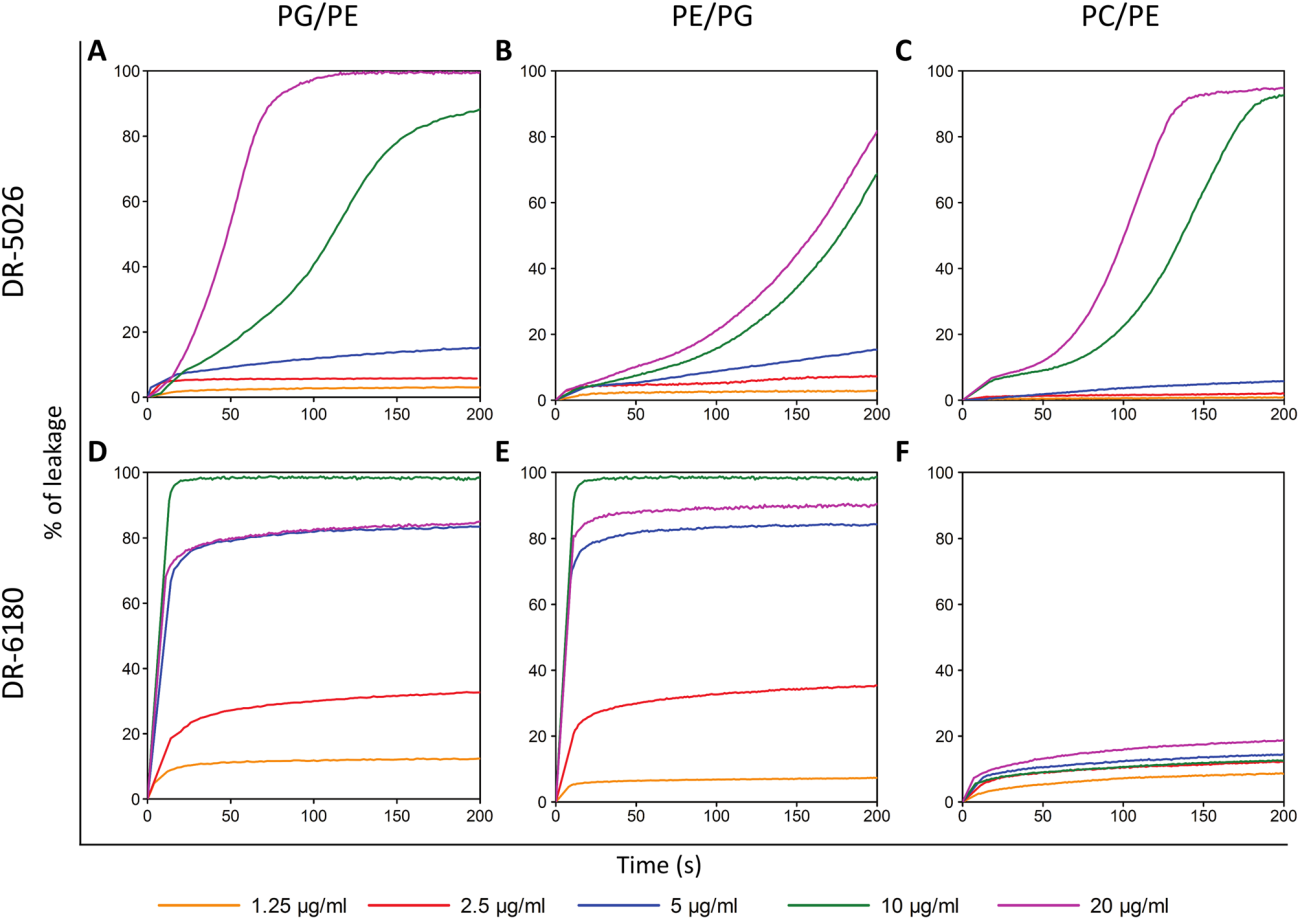
LPPO-induced leakage of carboxyfluorescein from liposomes. The curves show concentration dependence of LPPO I- (DR-5026, **A**–**C**) and LPPO II- (DR-6180, **D**–**F**) induced leakage from liposomes over time. The liposomes were composed of dioleyl-phospholipids in a 2:1 (w/w) ratio—PG/PE, PE/PG and PC/PE. The LPPO/phospholipid ratios were as follows: DR-5026—0.18/1, 0.36/1, 0.72/1, 1.43/1 and 2.86/1; DR-6180—0.14/1, 0.29/1, 0.58/1, 1.15/1 and 2.30/1. 100% leakage was achieved using 0.1% Triton X-100. Representative kinetics from at least three independent liposome preparations are shown.

**Figure 8:**
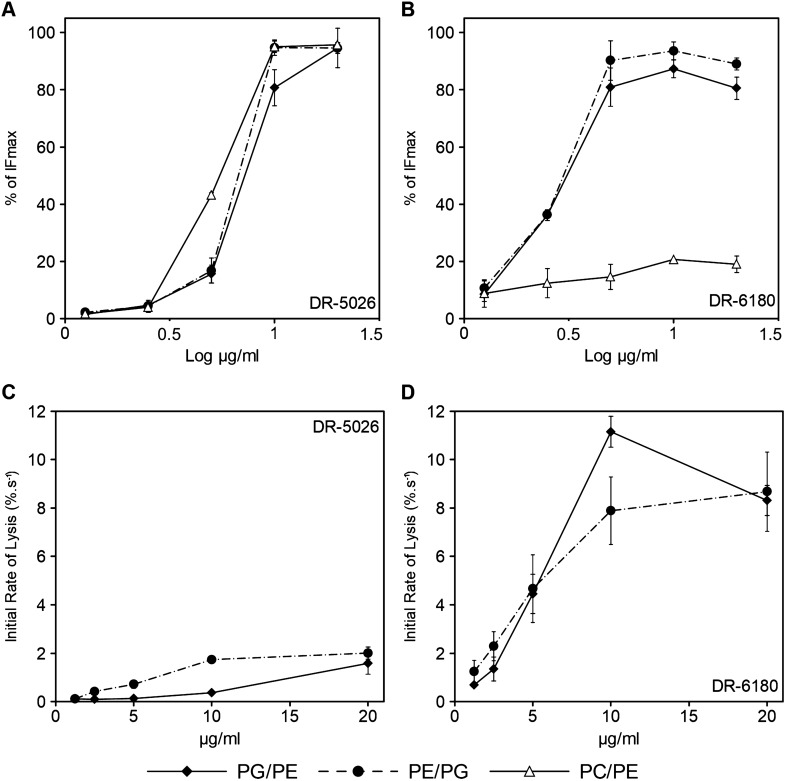
Concentration dependence of maximum LPPO-induced lysis and initial rate of lysis as a function of liposome membrane composition. (**A**,**B**) The data were taken from Fig. 7 and are plotted as the maximum reached in the plateau after exposure to a given LPPO concentration. (**C**,**D**) Dependence of the initial rate of lysis on LPPO concentration in liposomes differing in their lipid compositions. Average values from at least three liposome preparations and a standard deviation are shown.

The data in Fig. 7A–C show that LPPO I DR-5026 disrupted the PE/PG liposomes more slowly than the PG/PE ones. The leakage half-time (the time required after the addition of LPPO necessary to reach 50% of the maximum) was three times as long when using 20 µg/ml in PE/PG liposomes than in PG/PE ones. The DR-5026-induced lysis exhibited a dose-dependent behavior for all the phospholipid compositions tested. Of note, the kinetics up to 5 µg/ml were hyperbolic-like, and at a concentration of 10 and 20 µg/ml the curves exhibit a sigmoidal character. From Fig. 8A, which plots the concentration dependence of maximum LPPO-induced lysis as a function of liposome composition shown in Fig. 7, it is apparent that the inflection point corresponds to an even lower concentration than 10 µg/ml. This indicates the presence of two different modes of membrane permeabilization at lower and higher LPPO I concentration ranges. The effectivity of membrane permeabilization expressed as the initial rate of lysis (% s^−1^, Fig. 8C,D) and the maximum in the plateau is highest in PG/PE membranes. Leakage from the PE/PG and PC/PE liposomes was substantially slower and reached lower maximum values.

When using LPPO II DR-6180 (Fig. 7D–F), we observed higher permeabilizing activity in both PG/PE and PE/PG liposomes compared to DR-5026—the fluorescence intensity rose more steeply, and a concentration of just 10 µg/ml caused complete lysis. Surprisingly, the concentration of 20 µg/ml had a comparable effect to that of 5 µg/ml. In contrast to LPPO I DR-5026, the initial rates of lysis were much higher in both PG/PE and PE/PG liposomes (Fig. 8D) and the dose–response curves in Fig. 8B exhibited hyperbolic-like behavior. The overall extent of liposome lysis was the same for both bacteria-like liposomes; however, the DR-6180 activity changed substantially in the completely neutral membrane of PC/PE liposomes. Almost irrespective of the concentration used (Figs. 7F, 8B), the LPPO II-induced lysis was at most 20%.

### The presence of lipopolysaccharide decreases the effectiveness of LPPO-induced permeabilization

**Figure 9:**
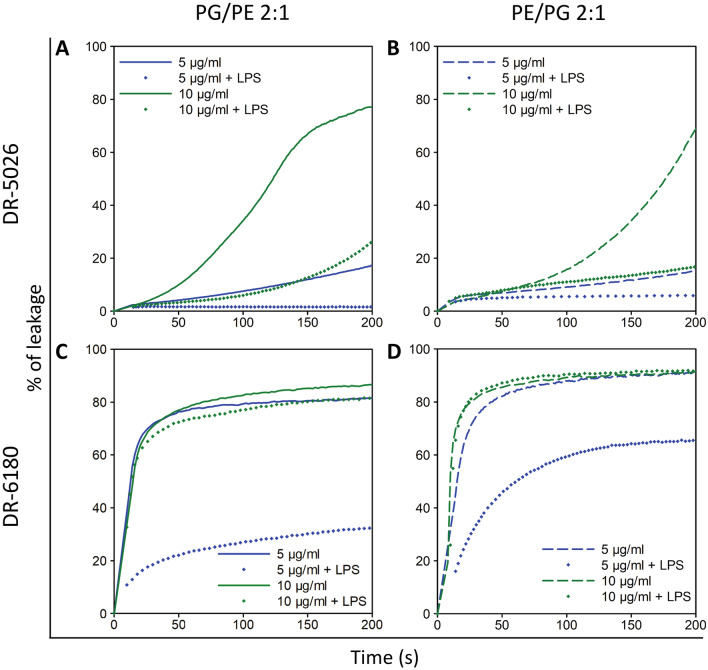
Effect of LPS on LPPO-induced leakage from liposomes. Carboxyfluorescein-loaded liposomes composed of dioleyl-phosphatidylglycerol (solid line) and diolelyl-phosphatidylethanolamine (dashed line) in ratios of 2:1 and 1:2 (w/w), respectively, which is the same as in Fig. 7A,B,D,E. Liposomes were preincubated with LPS (for details, see “Materials and methods”) and then exposed to LPPO I DR-5026 (**A**,**B**) and LPPO II DR-6180 (**C**,**D**) at the indicated concentrations. The resulting leakage curves (diamonds) are compared with those using liposomes without LPS treatment. The phospholipid concentration in liposome suspension was 10 µM. Representative kinetics from three independent liposome preparations are shown.

Finally, we investigated whether the presence of lipopolysaccharide (LPS) could influence LPPO permeabilizing activity in the membrane. We used the same PG/PE and PE/PG liposomes as in the previous experiment (Fig. 7) and incubated those with LPS prior to LPPO addition. In Fig. 9 we can see that LPS apparently hinders LPPO action. With LPPO II (Fig. 9C,D) at a concentration of 5 µg/ml, the degree of liposome leakage was markedly reduced—the effect was even more pronounced in the PG/PE (Fig. 9C) membrane than in PE/PG one (Fig. 9D). The presence of LPS decreased the maximum degree of PG/PE liposome lysis 2.5-fold. This effect however disappeared when the LPPO II concentration was increased to 10 µg/ml. In contrast, with LPPO I (Fig. 9A,B), we observed a decreased permeabilization efficiency of liposome suspension containing LPS using both concentrations of LPPO. In PG/PE liposomes (Fig. 9A) this effect exhibited a greater concentration dependency than in PE/PG ones (Fig. 9B). In PG/PE liposomes, the presence of LPS lowered the maximum degree of lysis (not shown) 12-fold and 2.5-fold when using 5 and 10 µg/ml of LPPO I, respectively. With PE/PG liposomes, the maximum degree of lysis dropped threefold and fivefold when using 5 and 10 µg/ml of LPPO I, respectively. The results of these experiments are thus in line with the data obtained using living cells.

### *Escherichia coli* with compromised outer membrane is susceptible to LPPO I action

All the above experiments showed that the membrane lipid composition, namely the presence of higher proportions of neutral lipids, and the presence of outer membrane are responsible for the ineffectiveness of LPPO I in *E. coli*. Thus we finally decided to employ *E. coli* imp4213 with a compromised outer membrane to assess its sensitivity to the action of LPPO. This strain bears an in-frame deletion of the imp gene encoding the essential outer membrane protein LptD, which is involved in LPS assembly^22,23^. This produces permeability defects of the outer membrane of the mutant *E. coli* strain. The values of the minimum inhibitory concentration clearly showed (Table 2) that in contrast to *E. coli* CCM 3954, which has an intact outer membrane, the strain imp4213 is highly sensitive to the action of LPPO I DR-5026, as its MIC dropped from > 256 to 8 µg/ml when compared with *E. coli* CCM 3954. With respect to LPPO I activity in the Gram-negative bacterium, this result confirmed the substantial role of the outer membrane as a physical barrier that complements the effect of LPS.

**Table 2:** Minimum inhibitory concentrations of LPPO I (DR-5026) and II (DR-6180).

	MIC µg/ml	
	DR-5026	DR-6180
*E. coli* CCM3954	> 256	2
*E. coli* imp4213	8	1

## Discussion

LPPOs are modular antimicrobials with promising potential for future use as alternatives to current antibiotics. They kill bacteria by disrupting their cytoplasmic membrane via pore formation^17,18^. First-generation LPPOs were found to be effective against Gram-positive bacteria. However, they do not exhibit any activity against Gram-negative bacteria. The modification of the imino-sugar module of the LPPO molecule and the increase in the number of positive charges gave rise to second-generation LPPOs which exhibit activity against both Gram-positive and Gram-negative bacteria. In this study, we aimed at elucidating the nature of the different effectiveness of LPPO I and LPPO II against Gram-positive and -negative bacteria. We studied the effect of LPPOs over a wide concentration range, using both in vitro approaches and in vivo studies with model bacteria. We mostly had to use LPPO concentrations well above their MICs, because lower concentrations only resulted in a weak effect. The possible reason for this was that it was necessary to adjust the LPPO concentration to the higher number of cells in our assays (~ 10^7^ cells/ml compared to MIC—~ 10^6^ cells/ml), resulting in a larger membrane area.

Both generations of LPPOs share the same mode of action—pore formation. The distribution of pore conductances is very broad, ranging from a few pS up to nS in 1 M KCl. In this respect, the pore-forming activity is comparable to other small pore-forming antimicrobials such as daptomycin, which forms pores in model membranes (according to recent papers^24–26^), or surfactin^27^. In some pores the unpredictable dynamics may point to the fact that certain LPPO complexes do not adopt exact pore stoichiometry or it can change rapidly in time. Possibly membrane phospholipids take part in the pore formation. With our instrumentation we cannot be sure about the instant conductance of the events with the fast current fluctuations but the average conductance should be close to the correct value. However, for pores with very short opening dwell time the recorded conductance might be lower than the real one. Nevertheless, the most frequent pore conductances do not differ much between the two LPPOs tested. Also, the concentration dependency shows the same pattern for both LPPOs—a twofold decrease in LPPO concentration leads to a several-fold decrease in the most frequent conductance states and a disappearance of the large conductance states with a pore conductance of several hundred pS. When we further lowered the LPPO concentration, the most frequent pore conductance states did not change. This suggests that LPPO pores are probably oligomeric with a varying number of monomers depending on LPPO concentration, and that a minimal conductance unit exists which most likely has a conductance of 20 pS.

Based on the known MICs for the model Gram-positive bacterium *B. subtilis* and the Gram-negative bacterium *E. coli* (Table 1), we tested the permeabilizing activity in living cells using the dyes PI and DiSC_3_(5). While an increase in PI generally signifies inner membrane permeabilization, the rise in DiSC_3_(5) fluorescence indicates membrane depolarization, i.e. that the membrane is permeable for small ions such as H^+^, K^+^ or Na^+^. The PI assay showed (Fig. 3) that the permeabilizing activity is concentration-dependent (although very weakly in *B. subtilis*). In line with very high MIC values for *E. coli*, this experiment proved that LPPO I DR-5026 is ineffective against *E. coli* cells. Of note, using LPPO II DR-6180, we observed different shapes of the kinetics traces for *B. subtilis* and *E. coli*. While the kinetics of PI entry into *B.* *subtilis* were steep and hyperbolic, kinetics with *E. coli* were slow and multiphasic, suggesting that several modes of membrane permeabilization occurred. We speculated that this might be caused by the different phospholipid composition of the cytoplasmic membrane (discussed further in the text) and different structure of the cell envelope. The presence of the outer membrane represents a barrier which the LPPO molecules must first overcome to reach their target and thus the permeabilizing process is delayed. In *E. coli* it took several minutes to reach the plateau, whereas in *B. subtilis* it was only several tens of seconds. We might speculate that at first LPPO II acts on *E. coli* at lower concentrations, forming smaller oligomers in the cytoplasmic membrane, and then when the molecules accumulate in the periplasmic space, the formation of the larger oligomers predominates and thus the rate of permeabilization increases.

The experiment with the probe DiSC_3_(5) confirmed that both LPPO I and II are capable of depolarizing the cytoplasmic membrane of the tested bacteria. With regard to LPPO type and model organism, the DiSC_3_(5) kinetics (Fig. 4) shared the same difference in their shape and time-course as the PI ones. Surprisingly, we also observed a slight depolarization of the cytoplasmic membrane of *E. coli* treated with LPPO I (DR-5026); however, the maximum effect induced by a concentration of 20 µg/ml was comparable to that of 5 µg/ml of LPPO II DR-6180. This means that, even in *E. coli*, some DR-5026 molecules must have entered the cytoplasmic membrane and formed small pores which allowed the passage of small ions. Nevertheless, these pores were not large enough to enable the passage of the probe PI, which has a molecular weight of 668 Da. However, such a small depolarization is not able to induce a sufficient effect on cellular metabolism to be observable as a decrease in MIC value.

Based on the results from PI and DiSC_3_(5) kinetics, we put forward several plausible reasons for the different susceptibility of *B. subtilis* and *E. coli* to LPPO I and II. We hypothesized that it may be caused by (i) the different structure of their cell envelopes, i.e. the presence of the outer membrane in *E. coli*, and/or by (ii) different lipid composition of the target cytoplasmic membrane. Generally, the activity of membrane-active antimicrobials depends on target membrane characteristics such as membrane lipid composition^20,28^ and membrane potential^29,30^. The mode of action of some antimicrobial peptides includes targeting specific lipids^31^ of the bacterial inner membrane. Therefore, modified lipid composition or decreased value of membrane potential may lead to a lowered antibiotic activity^24^ or even to antimicrobial resistance^32^. As for the membrane potential, in our experiments we did not observe any marked impact of the value of the target membrane potential on the activity of LPPO I or LPPO II (Fig. 5A,B). This is a highly desirable feature of an antimicrobial compound, as it means that persistent cells, which have a lower membrane potential^30^, are also susceptible to their antimicrobial effect, which we confirmed in the experiment shown in Fig. 5C.

To test the hypothesis that LPPO I molecules cannot traverse through the outer membrane, we performed an NPN assay (Fig. 6), which showed that LPPO I (DR-5026) only negligibly compromises the integrity of the outer membrane of living *E. coli* cells across the whole concentration range of 0.625–20 µg/ml. On top of that, leakage from liposomes with a composition that mimics the inner membrane phospholipid composition of Gram-positive, Gram-negative bacteria and the plasma membrane of eukaryotic cells showed that a different phospholipid composition substantially influences the permeabilizing potential of the tested LPPOs (Figs. 7, 8). The permeabilizing activity of LPPO II (DR-6180) was the same in liposomes with a different PG and PE ratio; however, it was substantially reduced in “eukaryotic” PC/PE liposomes (Fig. 8B) which is in line with the almost same MIC for *B.* *subtilis* and *E.* *coli* and LPPO II’s low hemolytic activity^18^. When using LPPO I DR-5026, we observed that the rate of leakage was generally lower than for DR-6180, with the exception of PC/PE liposomes, where higher DR-5026 concentrations also resulted in a massive leakage. This observation is at odds with the fact that DR-5026 had no detectable activity on normal primary cell viability and toxicity at the MIC concentrations^19^. We suggest that in living cells other lipids, sterols or non-lipid compounds of membrane may also affect LPPO activity. We also need to take into account the different LPPO/lipid ratio in cells and liposomes.

Importantly, the overall activity of DR-5026 decreased in liposomes mimicking the Gram-negative bacterial inner membrane. This suggests that the higher proportion of negatively charged PG in the Gram-positive-like membrane plays a key role in the interaction and/or pore formation by LPPO. Our results indicate that the affinity of LPPO II DR-6180 for PG is higher compared to LPPO I DR-5026. This might be due to the coulombic effect between the positive charges of LPPO and the negative charge of PG. Both DR-5026 and DR-6180 bear a positive charge (born by the tertiary amine and the nucleobase) which is attracted to the negative charge of PG. In addition, DR-6180 has two highly positive guanidinium groups which may result in higher electrostatic interaction between DR-6180 and PG compared to DR-5026. Thus the absence of PG in PC/PE liposomes may result in inefficacy of LPPO II in these types of bilayers.

LPS, which is part of the outer membrane of Gram-negative bacteria, hinders the activity of antimicrobial compounds^33^ by creating a barrier that is relatively impermeable to hydrophobic compounds^34^. This phenomenon was also confirmed in our leakage experiment with liposomes pre-incubated with LPS prior to the addition of LPPO (Fig. 9). The reduced activity was the most pronounced in PE/PG liposomes treated with DR-5026, or in other words the activity of DR-5026 was hindered the most substantially in these liposomes. We presume that in these LPS-liposomes two effects are combined—the presence of a lower proportion of negatively charged PG and the barrier function of LPS. The substantially reduced magnitude of DR-5026-induced leakage in LPS-PE/PG liposomes corroborated the results of the NPN assay in living *E. coli* cells, which showed that DR-5026 is unable to compromise the outer membrane. Finally, the role of the outer membrane in LPPO I ineffectiveness was further confirmed by the MIC value of DR-5026 against an *E. coli* imp4213 strain^22^ with compromised integrity of the outer membrane (due to a mutation in LPS assembly). Of note, the data showed that this strain was susceptible to LPPO I (Table 2).

The protective function of LPS is realized both by its hydrophilic densely packed oligosaccharide core with very low fluidity and by the hydrophobic hydrocarbon chain region^34^. The negatively charged phosphate groups on LPS are thought to be responsible for the interaction and binding of cationic AMPs^35^, which is a prerequisite for their subsequent insertion into the membrane. A recent study on the small AMP crabrolin showed that an increase in positive charges parallels an increased binding to LPS^36^. Hence, we propose that the substantially higher positive charge of LPPO II leads to a stronger electrostatic interaction with the negatively charged membrane. This interaction increases the probability that the hydrophobic part of LPPO II is anchored to the membrane, which results in higher antimicrobial activity against Gram-negative bacteria. The role of the number of positive charges on LPS binding and the antibacterial potential has been thoroughly studied using polymyxin B derivatives. Reduction in the number of positive charges of polymyxin B derivatives NAB7061 or NAB741 results in loss of any antibacterial activity; however, these derivatives potentiates activity of other antibiotics by reducing their MICs against Gram-negative pathogens^12^.

We might also speculate that not only the charge but also the fluidity of the outer membrane affects LPPO action as the mutant *E. coli* imp4213 strain which has reduced membrane stiffness^37^ become susceptible to LPPO I. In this mutant strain the presence of defective LPS molecules inhibits the insertion of many outer membrane proteins, which place is substituted with phospholipids. This results in a much higher permeability than the normal LPS/phospholipid bilayer^38^. All in all, although LPS is highly negatively charged and considered as the first target for cationic antimicrobial peptides, it can also serve as a barrier to prevent the insertion of AMPs into the inner phospholipid membrane due to its tight packing which hinders AMPs to traverse into the cytoplasmic membrane^33,39^. In conclusion, the inability of first-generation LPPO to exhibit antimicrobial activity against Gram-negative bacteria is due to several factors. We propose that the inability to overcome the outer membrane of Gram-negative bacteria might be the first factor explaining the inefficiency of LPPO I against Gram-negatives. LPPO I molecules probably interact with LPS and are incapable of disrupting the outer membrane, which in turn reduces their permeabilizing effect in the cytoplasmic membrane. After all, some of the LPPO I molecules finally reach their target, where they form pores through which small ions are able to pass, and the membrane is partially depolarized. However, the formation of larger pores that would lead to complete depolarization and cell death is probably limited by the absence of enough monomers. Moreover, the phospholipid composition of the target cytoplasmic membrane also reduces the efficiency of pore formation—the higher proportion of neutral lipids in *E. coli* leads to reduced membrane permeabilization. These effects add up, and might be the basis for LPPO I ineffectiveness in Gram-negative bacteria. The findings should be taken into account when designing new antimicrobial compounds.

## Methods

### Planar lipid bilayer experiments

A Teflon chamber was divided into two compartments connected by a circular aperture of approx. 0.5 mm in diameter. Planar lipid bilayers (black lipid membranes) were formed by painting a solution of 3% (w/v) 1,2-diphytanoyl-sn-glycero-3-phospho-(1′-rac-glycerol) (Avanti Polar Lipids) in *n*-decane-butanol (9:1, v/v) across the hole. Both compartments contained 1 ml of 1 M KCl, 10 mM Tris, pH 7.4. The temperature was kept at 25 °C. LPPO was added to the *cis* side of the membrane at a concentration of 5, 2.5 or 1.25 µg/ml, respectively. The membrane current was measured with Ag/AgCl electrodes with a constant applied voltage of 50 mV. The current signal was amplified with an LCA-200-100GV amplifier (Femto) and digitized with a KPCI-3108 card (Keithley). The signal was processed with QuB software^40^. In order to prevent the bin edge effect the histograms of single-pore conductance were created using kernel density estimation (rectangular kernel with a 30 pS width). For a more detailed explanation of kernel density estimation please see the Supplementary information. Raw experimental data from these conductivity measurements are available at Zenodo repository (http://dx.doi.org/10.5281/zenodo.4694562).

### Membrane permeabilization assay

The assay was performed as described previously^41^. Bacterial cells of *Bacillus subtilis* (168, *trp*^+^, BaSysBio^42^) and *Escherichia* *coli* CCM 3954 were grown aerobically in LB medium at 37 °C to the mid log phase (OD_450nm_ ~ 0.5). The cells were harvested (8000*g*, 25 °C, 10 min), washed, and resuspended (final OD_450nm_ ~ 0.2) in a buffer containing 10 mM HEPES (pH 7.2), 0.5% glucose and 10 μM propidium iodide (PI, Invitrogen). This cell suspension was readily used for the assay without further incubation. LPPOs were added to 2 ml of bacterial suspension in a 10 × 10-mm quartz cuvette and propidium iodide (PI) uptake into cells (indicating membrane permeabilization) was monitored as the increase in fluorescence intensity (excitation at 515 nm, emission at 620 nm with bandpass 5 and 5 nm, respectively) at 25 °C using a FluoroMax-3 spectrofluorometer (Jobin Yvon, Horriba). We used optical filters to suppress light scattered by the cells (Omega Optical filters 3RD500-530 and 3RD570LP in excitation and emission paths, respectively). The bacterial suspension was continuously stirred with a magnetic stirrer during the measurements. As a positive control for cell permeabilization, 10 μM melittin (Sigma) was added to the cuvette, whereas the addition of the buffer alone served as a negative control. The presented data are the recorded intensities without background subtraction. LPPOs interaction with PI in solution resulted in maximum 5% change in fluorescence intensity (Supplementary Fig. S3).

To adjust the desired value of electrical membrane potential ΔΨ on bacterial cells, we used a common method utilizing the selective K^+^ ionophore valinomycin and a known K^+^ gradient across the membrane^43,44^. The Nernst equation was used to calculate the K^+^ concentration in the buffer (K^+^_out_) from the desired ΔΨ value, a mean intracellular K^+^ concentration (K^+^_in_) of 300 mM^45^ was used. The calculated (K^+^_out_) values were used to prepare buffers with different KCl concentrations (Table 3) and containing 10 mM HEPES (pH 7.2), 0.5% glucose. To compensate for the different ionic strengths of the buffers, NaCl was added to reach a final electrolyte concentration of 300 mM. After the addition of 4 µM valinomycin (Sigma) to the bacteria, the electrical current across their membrane was induced by selective K^+^ transport, which sets up a diffusion potential (Supplementary Fig. S4) within a few minutes. The assay was carried out in a 96-well plate using a MicroMax 384 Microwell-Plate Reader, each well contained 200 µl of the cell suspension. The increase in PI fluorescence intensity was measured at 25 °C using a FluoroMax-3 spectrofluorometer (Jobin Yvon, Horriba) using the same settings as described above (with the exception of bandpasses, which were both 15 nm in this case). Due to the delay in the preparation of samples on the well plate, the first points of the presented kinetics are missing. Before the addition of a tested compound, the initial intensities were comparable in all data sets.

**Table 3:** Demanded ΔΨ equilibrium potentials and corresponding calculated [K^+^].

ΔΨ (mV)	[K^+^]_in_ (mM)	[K^+^]_out_ (mM)
0	300	300
− 50	300	46
− 100	300	7

### Membrane potential measurement

For observing the changes in membrane potential in *B. subtilis* and *E. coli*, we used the voltage-sensitive dye DiSC_3_(5) (1 μM, 1% DMSO, Sigma), which accumulates in hyperpolarized membranes, where its fluorescence is quenched. The probe released from the depolarized membranes exerts increased fluorescence intensity^44^. Bacteria were grown aerobically in LB medium at 37 °C to the mid log phase (OD_450nm_ ~ 0.5). The cells were harvested (8000*g*, 25 °C, 10 min), washed, and resuspended in a buffer containing 10 mM HEPES (pH 7.2), 0.5% glucose and 1 μM DiSC_3_(5) (Sigma)ml to final OD_450nm_ ~ 0.2 (corresponding to ~ 2 × 10^7^ cells/ml). The incubation took 90 min in the dark and the cell suspension was continuously stirred. These labeling conditions ensured stable fluorescence signal. LPPOs were added to 2 ml of this suspension in a 10 × 10-mm quartz cuvette, and the increase in DiSC_3_(5) fluorescence intensity was measured at 25 °C using a FluoroMax-3 spectrofluorometer (Jobin Yvon, Horriba). Excitation and emission wavelengths were set to 600 nm and 670 nm, respectively (both bandpasses of 4 nm). Optical filters (Omega Optical filters RPB590-610 and RPE650LP in the excitation and emission paths, respectively) were used to suppress light scattered by the cells. As a positive control for membrane depolarization, 10 μM melittin (Sigma) was added to the cuvette, whereas addition of the buffer alone served as a negative control. LPPOs interaction with DiSC_3_(5) in solution resulted in ~ 10% change in fluorescence intensity (Supplementary Fig. S3). The presented data are the recorded intensities without background subtraction.

### Persister killing assay

The experiment was performed as described by Grassi et al.^46^ using *E. coli* CCM 3954. Carbonyl cyanide *m*-chlorphenylhydrazone (CCCP, Sigma-Aldrich, United States) was diluted in DMSO (stock solution 40 mg/ml) and stored at − 20 °C. Minimum inhibitory concentrations of DR-6180 and colistin (Sigma-Aldrich, United States) were determined (as described above in Determination of MIC values) and concentrations corresponding to MIC and 5xMIC were used in the experiment.

The bacterial suspension was cultivated overnight in MH broth at 35 ± 1 °C with shaking. 1 ml of suspension was transferred into a microtube and incubated for 3 h at 35 ± 1 °C with 10 μl of CCCP (200 μg/ml). After the treatment, the bacteria were washed twice in saline solution (0.9% w/v NaCl) at 1700×*g* for 10 min and resuspended in saline at a final density of 5 × 10^8^ CFU/ml.

To evaluate the activity of LPPO II DR-6180 and colistin against CCCP-induced persisters, CCCP-treated and untreated bacteria were diluted in PBS (phosphate-buffered saline) supplemented with 1% MH broth to a final density of 10^6^ CFU/ml. After 3 h of incubation with gentle shaking at 35 ± 1 °C, samples were exponentially diluted and inoculated on MH agar. After additional incubation for 24 h at 35 ± 1 °C, the bacterial cells were counted to determine CFU/ml.

### Outer membrane integrity assays

The enhanced permeability of the outer membrane induced by LPPO was determined as the increase in fluorescence of the probe 1-*N*-phenylnapthylamine (NPN, Sigma-Aldrich). When the outer membrane of the cells is compromised, NPN can access the periplasmic space and bind the phospholipids, which increases its fluorescence^47,48^. Bacterial cells of *E. coli* CCM 3954 were grown to the mid-log phase (OD_450nm_ ~ 0.5), harvested (8000*g*, 25 °C, 10 min), washed, and resuspended (final OD_450nm_ ~ 0.2) in a buffer containing 10 mM HEPES (pH 7.2), 0.5% glucose and 10 μM NPN. This cell suspension was readily used for the assay without further incubation. LPPOs were added to 2 ml of bacterial suspension in a 10 × 10-mm quartz cuvette, and the increase in fluorescence intensity of NPN was monitored over time (excitation at 350 nm, emission at 420 nm with bandpass 5 and 5 nm, respectively) at 25 °C using a FluoroMax-3 spectrofluorometer (Jobin Yvon, Horriba). The representative kinetics of NPN uptake are shown in Supplementary Fig. S5. The bacterial suspension was continuously stirred with a magnetic stirrer during the measurements. The data are presented as the percentage of NPN uptake in the presence of LPPO relative to the maximum induced by 100 μM polymyxin B (IF_max_, Sigma-Aldrich) − % NPN uptake = (IF_LPPO_ − IF_0_)/(IF_max_ − IF_0_) × 100, where IF_LPPO_ is the fluorescence intensity in the plateau phase after the addition of LPPO, and IF_0_ is the fluorescence of *E.* *coli* cells in the buffer before the addition of LPPO. LPPOs interaction with NPN in solution resulted in ~ 5–15% change in fluorescence intensity (Supplementary Fig. S3).

### Liposome preparation

Liposomes were prepared using the method that we described previously^21^. 1,2-dioleoyl-sn-glycero-3-phospho-(1′-rac-glycerol) (DOPG), 1,2-dioleoyl-sn-glycero-3-phosphoethanolamine (DOPE) and 1,2-dioleoyl-sn-glycero-3-phosphocholine (DOPC) were purchased from Avanti Polar Lipids. Liposomes to be used in the carboxyfluorescein leakage assay were prepared by mixing the appropriate amounts of lipids (1 mg/ml) in chloroform. The solvent was evaporated in vacuo, obtaining a thin film on the walls of a glass tube. The hydration procedure in a buffer containing 50 mM 5(6)-Carboxyfluorescein (CF), 5 mM HEPES pH 7.4 lasted for 90 min at 40 °C, being interrupted by thorough vortex shaking to form multilamellar vesicles. Large unilamellar vesicles (LUVs) were prepared by repeated extrusion of the multilamellar vesicles through 100 nm polycarbonate filters (Whatman) using an extruder (Avanti Polar Lipids). Liposomes were separated from nonencapsulated dye by gel filtration on Sephadex G-50 using 100 mM NaCl, 0.5 mM Na_2_EDTA and 5 mM HEPES, pH 7.4 as elution buffer. The liposome suspension was diluted in the same buffer to give a final phospholipid concentration of 10 µM (according to the assessed content of inorganic phosphate).

### Liposome leakage assay

The leakage of liposome contents was evaluated by the increase in fluorescence intensity due to CF release into the milieu^21^ after adding LPPO solution to the final concentration indicated in the respective graph legends. The concentrations used in this assay are not comparable to the concentrations used in the cells since it is unclear how much of the compound would reach the inner membrane and how much is retained in the outer membrane and/or cell wall. Thus the compound/lipid ratios in cell cultures cannot be reasonably estimated.

To test the effect of the presence of lipopolysaccharide (LPS) on LPPO-induced liposome leakage, liposome suspension (10 µM inorganic phosphate concentration) was incubated with 20 μg/ml of lipopolysaccharide (LPS from *E. coli* O111:B4, Sigma-Aldrich) for 10 min prior to LPPO addition, thus the lipid-LPS ratio was 2:5 (w/w)^49^. The maximum increase in CF fluorescence (*F*_max_) was induced by lysing the vesicles with 0.1% (v/v) Triton X-100. Fluorescence intensity was monitored over time (excitation at 480 nm, emission at 515 nm) at 25 °C using a FluoroMax-3 spectrofluorometer (Jobin Yvon, Horriba). The following equation was used to calculate the percent of CF leakage: %CF leakage = [(*F* − *F*_0_)/(*F*_max_ − *F*_0_)] × 100, where *F* is the actual fluorescence intensity and *F*_0_ is the fluorescence intensity before the addition of the tested compound. Representative results from at least three individual experiments are shown.

### Determination of MIC values

The antimicrobial activity of the tested compounds against *E. coli* imp4213, in which the mutation in the imp gene causes a higher permeability of the outer membrane^23^, was assessed using the microdilution method to determine the minimum inhibitory concentration (MIC)^50^. Disposable microtitration plates were used for the tests. The compounds were diluted in MH broth (Himedia) to yield a concentration range between 256 and 1 µg/ml. The plates were inoculated with a standard amount of the tested microbe—the inoculum density in each well was equal to 10^6^ CFU/ml. The MIC was read after 24 h of incubation at 35 °C as the minimum inhibitory concentration of the tested substance that inhibited the growth of the bacterial strains.

## Supplementary Information

Supplementary Information.

## Data Availability

A custom made Perl script used to generate histograms of single-pore conductance presented in Fig. 2 is provided in Supplementary information.

## References

[1] Scott WR, Tew NG (2017). Mimics of host defense proteins; strategies for translation to therapeutic applications. Curr. Top. Med. Chem..

[2] Lei J (2019). The antimicrobial peptides and their potential clinical applications. Am. J. Transl. Res..

[3] Chen CH, Lu TK (2020). Development and challenges of antimicrobial peptides for therapeutic applications. Antibiotics.

[4] Giuliani A (2008). Antimicrobial peptides: Natural templates for synthetic membrane-active compounds. Cell. Mol. Life Sci..

[5] Matsuzaki K (2009). Control of cell selectivity of antimicrobial peptides. Biochim. Biophys. Acta Biomembr..

[6] Oh D (2014). Antibacterial activities of amphiphilic cyclic cell-penetrating peptides against multidrug-resistant pathogens. Mol. Pharm..

[7] Kustanovich I, Shalev DE, Mikhlin M, Gaidukov L, Mor A (2002). Structural requirements for potent versus selective cytotoxicity for antimicrobial dermaseptin S4 derivatives. J. Biol. Chem..

[8] Boto A, De La Lastra JMP, González CC (2018). The road from host-defense peptides to a new generation of antimicrobial drugs. Molecules.

[9] Thaker HD, Cankaya A, Scott RW, Tew GN (2013). Role of amphiphilicity in the design of synthetic mimics of antimicrobial peptides with gram-negative activity. ACS Med. Chem. Lett..

[10] Snyder S, Kim D, McIntosh TJ (1999). Lipopolysaccharide bilayer structure: Effect of chemotype, core mutations, divalent cations, and temperature. Biochemistry.

[11] Bertani B, Ruiz N (2018). Function and biogenesis of lipopolysaccharides. EcoSal Plus.

[12] Vaara M (2019). Polymyxin derivatives that sensitize gram-negative bacteria to other antibiotics. Molecules.

[13] Brown P, Dawson MJ (2017). Development of new polymyxin derivatives for multi-drug resistant Gram-negative infections. J. Antibiot..

[14] Vaara M, Vaara T (1983). Sensitization of Gram-negative bacteria to antibiotics and complement by a nontoxic oligopeptide. Nature.

[15] Koh JJ (2015). N-Lipidated peptide dimers: Effective antibacterial agents against gram-negative pathogens through lipopolysaccharide permeabilization. J. Med. Chem..

[16] Rounds T, Straus SK (2020). Lipidation of antimicrobial peptides as a design strategy for future alternatives to antibiotics. Int. J. Mol. Sci..

[17] Panova N (2015). Insights into the mechanism of action of bactericidal lipophosphonoxins. PLoS ONE.

[18] Seydlová G (2017). Lipophosphonoxins II: Design, synthesis, and properties of novel broad spectrum antibacterial agents. J. Med. Chem..

[19] Rejman D (2011). Lipophosphonoxins: New modular molecular structures with significant antibacterial properties. J. Med. Chem..

[20] Epand RM, Epand RF (2011). Bacterial membrane lipids in the action of antimicrobial agents. J. Pept. Sci..

[21] Uttlová P (2016). Bacillus subtilis alters the proportion of major membrane phospholipids in response to surfactin exposure. Biochim. Biophys. Acta Biomembr..

[22] Caetano T, Krawczyk JM, Mösker E, Süssmuth RD, Mendo S (2011). Lichenicidin biosynthesis in *Escherichia coli*: licFGEHI immunity genes are not essential for lantibiotic production or self-protection. Appl. Environ. Microbiol..

[23] Braun M, Silhavy TJ (2002). Imp/OstA is required for cell envelope biogenesis in *Escherichia coli*. Mol. Microbiol..

[24] Seydlová G, Sokol A, Lišková P, Konopásek I, Fišer R (2019). Daptomycin pore formation and stoichiometry depend on membrane potential of target membrane. Antimicrob. Agents Chemother..

[25] Gray DA, Wenzel M (2020). More than a pore: A current perspective on the in vivo mode of action of the lipopeptide antibiotic daptomycin. Antibiotics.

[26] Zuttion F (2020). High-speed atomic force microscopy highlights new molecular mechanism of daptomycin action. Nat. Commun..

[27] Pinkas D (2020). Bacillus subtilis cardiolipin protects its own membrane against surfactin-induced permeabilization. Biochim. Biophys. Acta Biomembr..

[28] Song C, de Groot BL, Sansom MSP (2019). Lipid bilayer composition influences the activity of the antimicrobial peptide dermcidin channel. Biophys. J..

[29] Damper PD, Epstein W (1981). Role of the membrane potential in bacterial resistance to aminoglycoside antibiotics. Antimicrob. Agents Chemother..

[30] Benarroch JM, Asally M (2020). The microbiologist’s guide to membrane potential dynamics. Trends Microbiol..

[31] Johnston CW (2016). Assembly and clustering of natural antibiotics guides target identification. Nat. Chem. Biol..

[32] Hachmann AB (2011). Reduction in membrane phosphatidylglycerol content leads to daptomycin resistance in *Bacillus subtilis*. Antimicrob. Agents Chemother..

[33] Papo N, Shai Y (2005). A molecular mechanism for lipopolysaccharide protection of gram-negative bacteria from antimicrobial peptides. J. Biol. Chem..

[34] Snyder DS, McIntosh TJ (2000). The lipopolysaccharide barrier: Correlation of antibiotic susceptibility with antibiotic permeability and fluorescent probe binding kinetics. Biochemistry.

[35] Rosenfeld Y, Sahl HG, Shai Y (2008). Parameters involved in antimicrobial and endotoxin detoxification activities of antimicrobial peptides. Biochemistry.

[36] Cantini Y (2020). Effect of positive charges in the structural interaction of crabrolin isoforms with lipopolysaccharide. J. Pept. Sci..

[37] Rojas ER (2018). The outer membrane is an essential load-bearing element in Gram-negative bacteria. Nature.

[38] Nikaido H (2005). Restoring permeability barrier function to outer membrane. Chem. Biol..

[39] Allende D, McIntosh TJ (2003). Lipopolysaccharides in bacterial membranes act like cholesterol in eukaryotic plasma membranes in providing protection against melittin-induced bilayer lysis. Biochemistry.

[40] Nicolai C, Sachs F (2013). Solving ion channel kinetics with the QuB software. Biophys. Rev. Lett..

[41] Seydlová G (2017). Lipophosphonoxins II: Design, synthesis, and properties of novel broad spectrum antibacterial agents. J. Med. Chem..

[42] Nicolas P (2012). Condition-dependent transcriptome reveals high-level regulatory architecture in *Bacillus subtilis*. Science (80-)..

[43] Klapperstück T, Glanz D, Klapperstück M, Wohlrab J (2009). Methodological aspects of measuring absolute values of membrane potential in human cells by flow cytometry. Cytom. Part A.

[44] te Winkel JD, Gray DA, Seistrup KH, Hamoen LW, Strahl H (2016). Analysis of antimicrobial-triggered membrane depolarization using voltage sensitive dyes. Front. Cell Dev. Biol..

[45] Whatmore AM, Chudek JA, Reed RH (1990). The effects of osmotic upshock on the intracellular solute pools of *Bacillus subtilis*. J. Gen. Microbiol..

[46] Grassi L (2017). Generation of persister cells of *Pseudomonas aeruginosa* and *Staphylococcus aureus* by chemical treatment and evaluation of their susceptibility to membrane-targeting agents. Front. Microbiol..

[47] Helander IM, Mattila-Sandholm T (2000). Fluorometric assessment of Gram-negative bacterial permeabilization. J. Appl. Microbiol..

[48] Nikaido H (2003). Molecular basis of bacterial outer membrane permeability revisited. Microbiol. Mol. Biol. Rev..

[49] Singh S, Kasetty G, Schmidtchen A, Malmsten M (2012). Membrane and lipopolysaccharide interactions of C-terminal peptides from S1 peptidases. Biochim. Biophys. Acta Biomembr..

[50] European Committee for Antimicrobial Susceptibility Testing (EUCAST) of the European Society of Clinical Microbiology and Infectious Diseases (ESCMID). Determination of minimum inhibitory concentrations (MICs) of antibacterial agents by broth dilution. *Clin. Microbiol. Infect.* **9**, ix–xv (2003).

