# Supplementary material for: Outer membrane and phospholipid composition of the target membrane affect the antimicrobial potential of first- and second-generation lipophosphonoxins
Source: Sci Rep. 2021 May 17;11:10446. doi: 10.1038/s41598-021-89883-0 (PMC8129119; doi:10.1038/s41598-021-89883-0)
Supplement: Supplementary file 1 — Supplementary Information. [file 41598_2021_89883_MOESM1_ESM.docx]

# Supplementary information

# Outer membrane and phospholipid composition of the target membrane affect the antimicrobial potential of first- and second-generation lipophosphonoxins

Klára Látrová, Noemi Havlová, Renata Večeřová, Dominik Pinkas, Kateřina Bogdanová, Milan Kolář, Radovan Fišer, Ivo Konopásek, Duy Dinh Do Pham, Dominik Rejman, Gabriela Mikušová

**
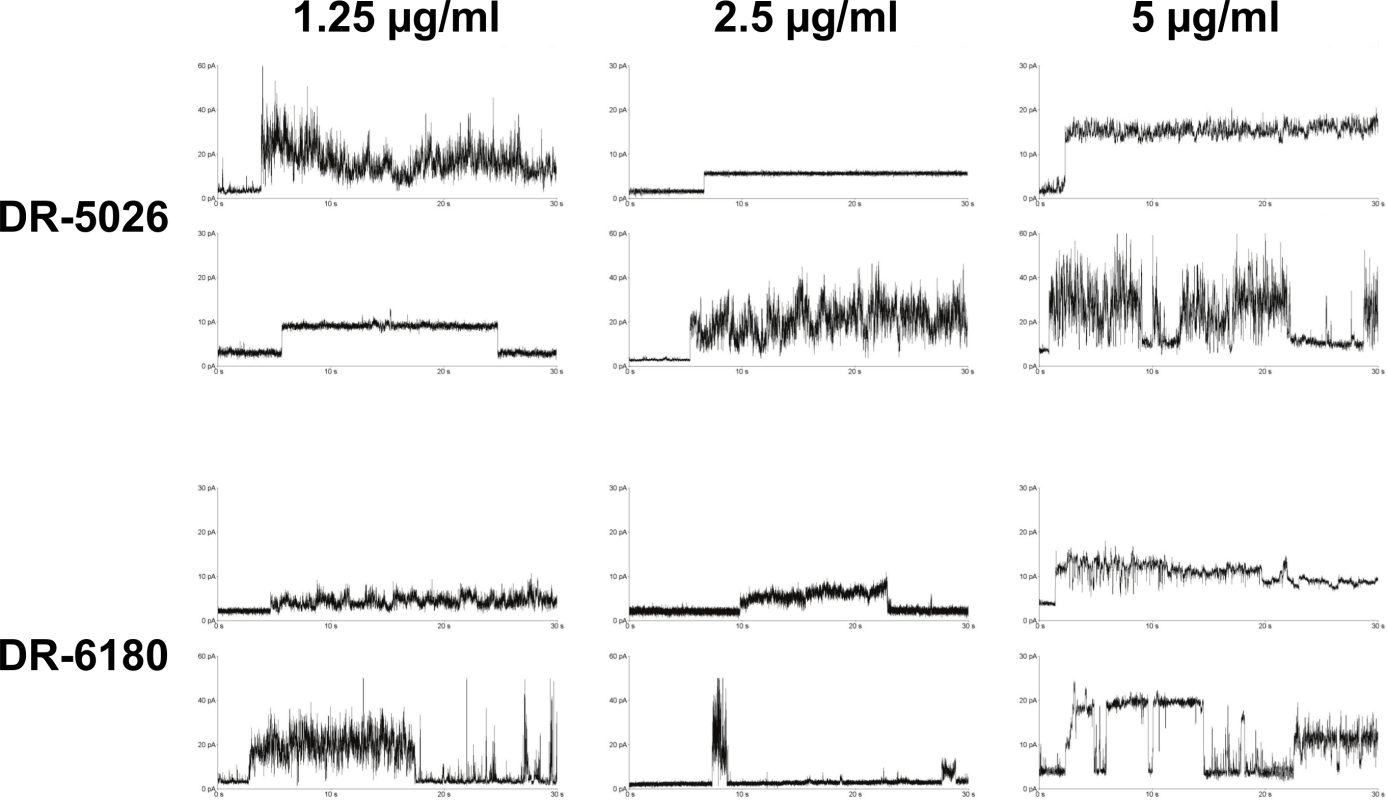
**

**Supplementary Figure S1. Representative current traces of LPPO pore formation**

Representative ion current recordings of LPPO I DR-5026 and LPPO II DR-6180 in 1M KCl, 10mM Tris, pH 7.4 with 50 mV voltage applied on diphytanoylphosphatidylglycerol membranes. Both LPPOs formed pores with high current noise and the typical current recordings did not change with the LPPO concentration used.


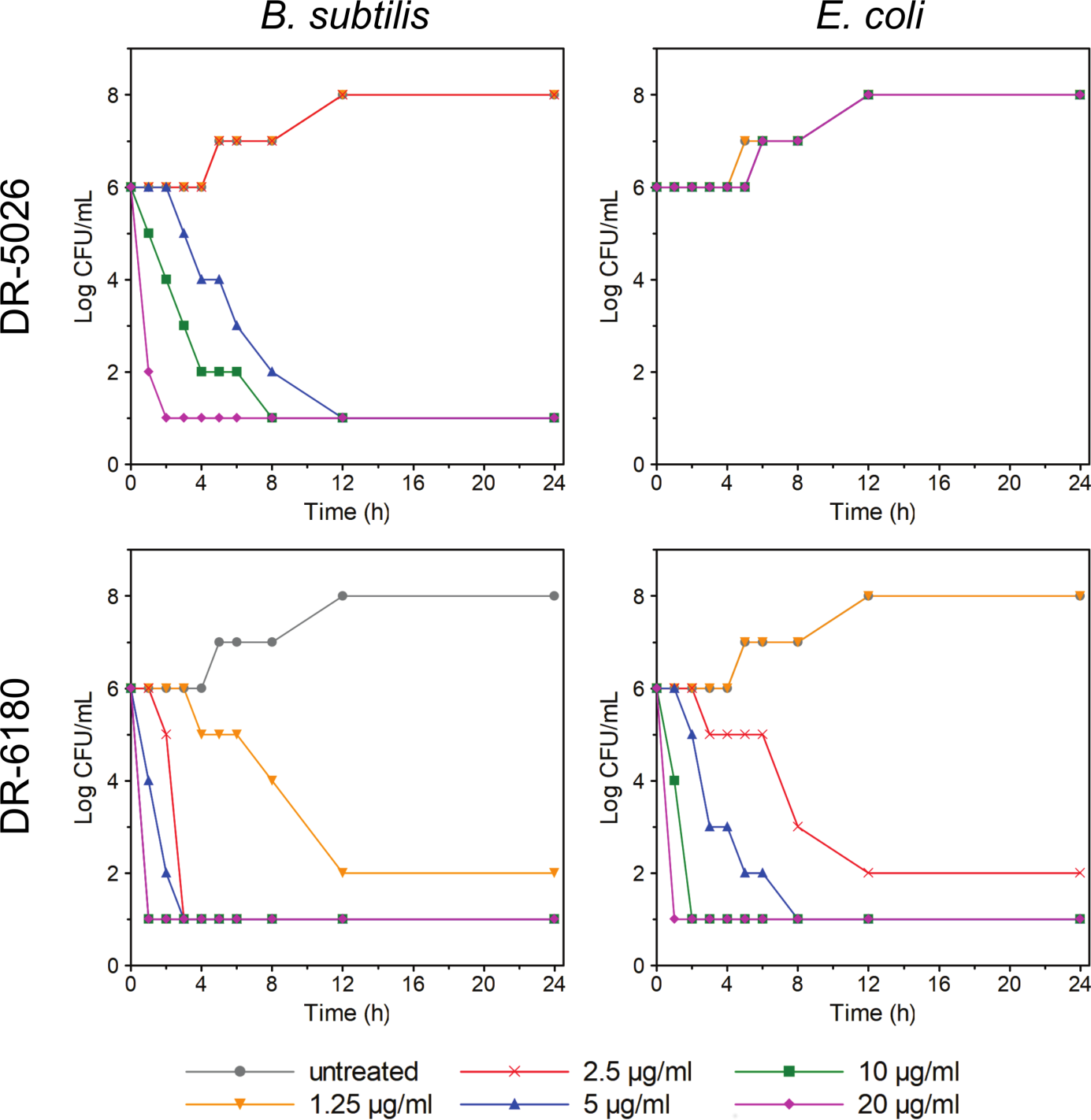


**Supplementary Figure S2. Determination of growth kinetics in the presence of LPPOs**

Determination of growth kinetics in the presence of tested LPPOs was performed in a microtiter plate. LPPOs DR-5026 and DR-6180 were diluted to concentrations of 20, 10, 5, 2.5 and 1.25 mg/l in MH broth inoculated with *Bacillus subtilis* and *Escherichia coli*. The initial concentration of the bacterial inoculum was 10^6^ CFU/ml. The prepared mixtures of LPPOs and bacteria were incubated for 24 hours at 35 ± 1 °C. At time intervals of 0 h, 1 h, 2 h, 3 h, 4 h, 5 h, 6 h, 8 h, 12 h and 24 h, a volume of 10 µl of the suspension was diluted and spread on MH agar. After incubation for 24 hours at 35 ± 1 °C, bacterial growth was evaluated and time-kill curves were created. For a better readability, each zero CFU value was arbitrarily transformed to Log CFU/ml value 1.


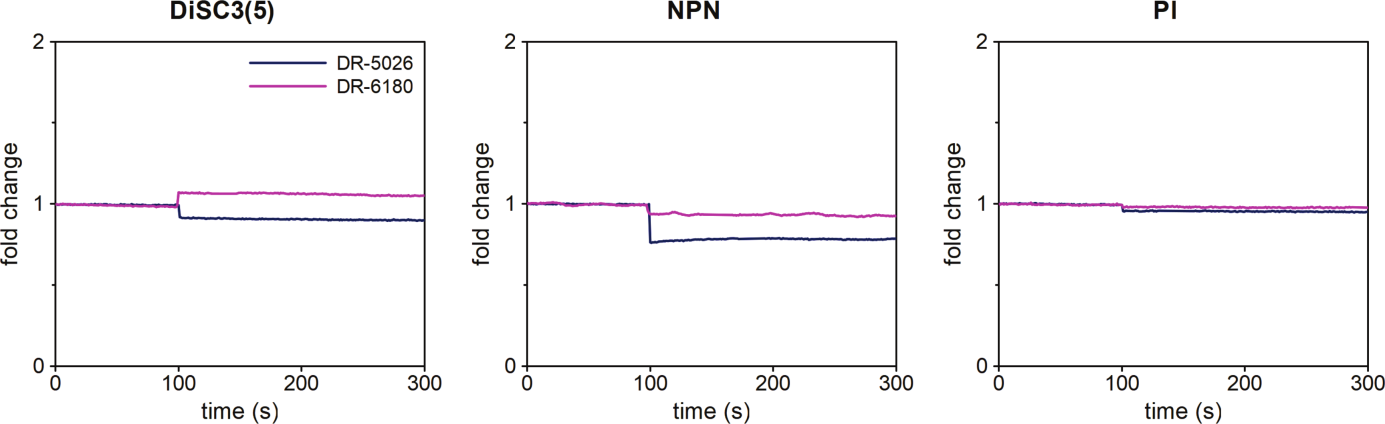


**Supplementary Figure S3. Interaction of LPPOs with fluorescence dyes**

Fold change of DiSC_3_(5), NPN and PI fluorescence intensity after LPPO addition. LPPO were added in the concentration of 20 µg/ml to the dye solution (in the concentration of 1, 10 and 10µM for DiSC_3_(5), NPN and PI, respectively) in 10mM HEPES buffer with 0.5% glucose (pH 7.2) at time point 100 s. These conditions mimicked the conditions of the respective in vitro experiments. The relative change in fluorescence intensity was ~5-15 %. The experiment was performed in a 10 × 10-mm quartz cuvette containing 2 ml of dye solution. The increase in DiSC_3_(5), NPN and PI fluorescence intensity was measured at 25 °C using a FluoroMax-3 spectrofluorometer (Jobin Yvon, Horriba). Excitation and emission wavelengths were set to: DiSC_3_(5) - 600 and 670 nm, respectively (both bandpasses of 3 nm), NPN – 350 and 420 nm, respectively (both bandpasses of 3 nm), PI – 515 and 620 nm, respectively (both bandpasses of 5 nm). In the case of DiSC_3_(5) optical filters (Omega Optical filters RPB590-610 and RPE650LP) were used. The results of this control experiment show no substantial artifacts of the in vitro assays.

**
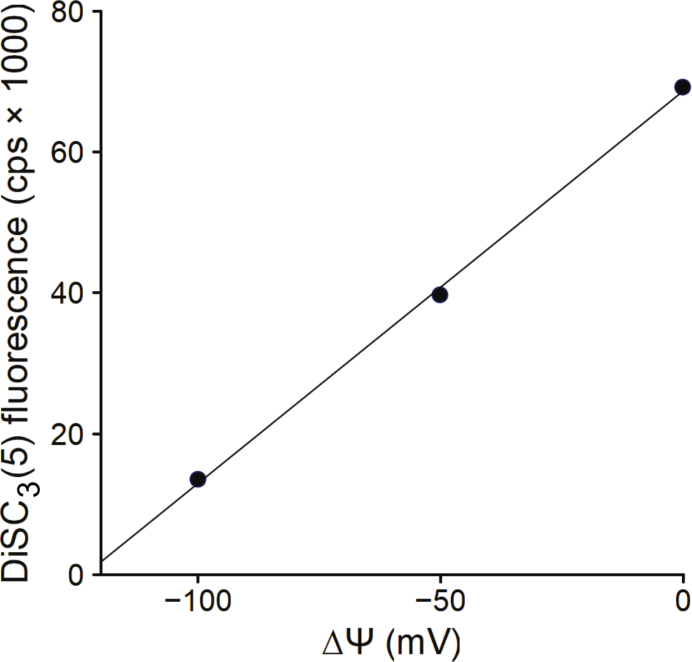
**

**Supplementary Figure S4. Calibration of DiSC_3_(5) assay**

Representative calibration of DiSC_3_(5) assay using *B. subtilis* cells (300 mM K^+^_in_) resuspended in buffers with various K^+^_out_ concentrations – 300 mM, 46 mM and 7 mM. The desired value of membrane potential ∆Ψ (0, -50 and -100 mV) was adjusted by the addition of 4 µM valinomycin^1^.

1. te Winkel, J. D., Gray, D. A., Seistrup, K. H., Hamoen, L. W. & Strahl, H. Analysis of Antimicrobial-Triggered Membrane Depolarization Using Voltage Sensitive Dyes. *Front. Cell Dev. Biol.* **4**, 29 (2016).


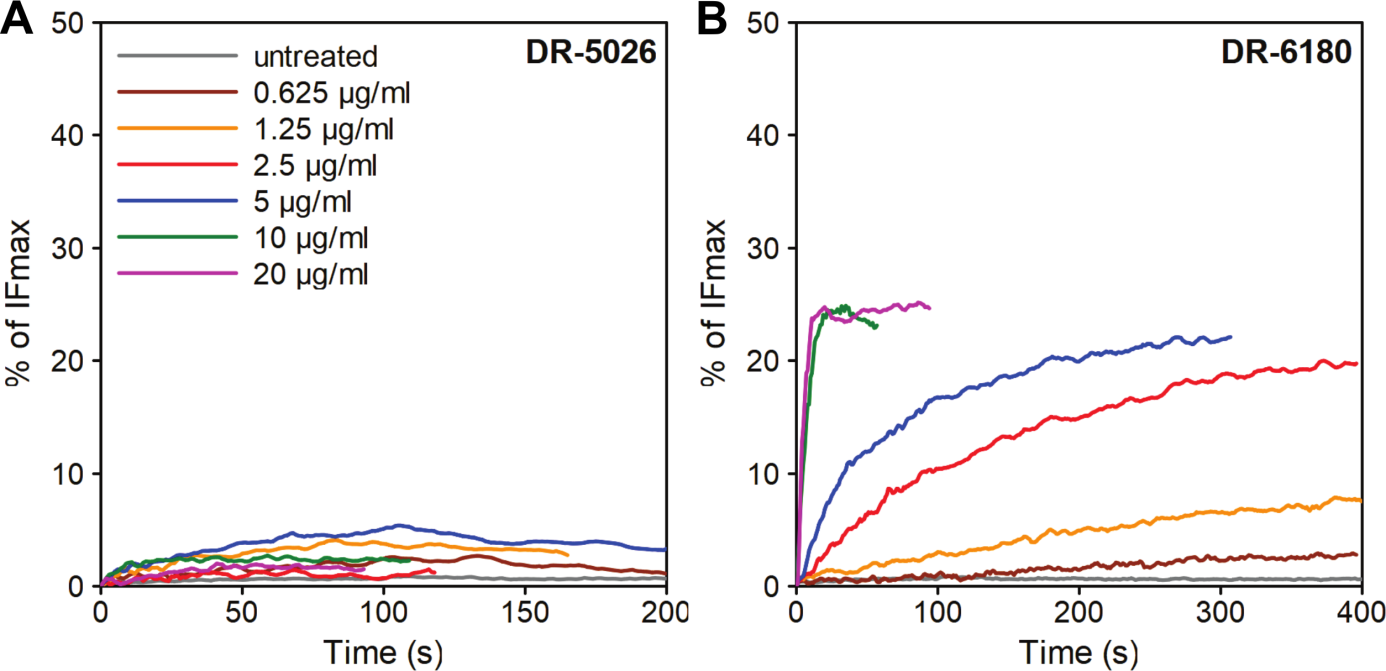


**Supplementary Figure S5. Kinetics of outer membrane permeabilization induced by LPPOs**

The integrity of the outer membrane was assessed by measuring the increase in fluorescence intensity due to NPN uptake by *E. coli* cells with a compromised outer membrane induced by LPPO I (A, DR-5026) and LPPO II (B, DR-6180). 100% outer membrane permeabilization (IF max) was achieved using 100µM polymyxin B. Representative kinetics from at least three independent experiments are shown.

**Kernel density estimation**

We select individual single-pore events from the recordings manually using QuB software which is then used for conductance quantifications. These conductance values are transformed into histogram or kernel density estimation (KDE). When presenting and comparing the most frequent conductance state of LPPO pores we use KDE instead of standard conductance histograms. When creating conventional histograms one usually sorts the data into individual categories, so called bins. The height of the bar in the histogram corresponds to the number of event observation within each bin. This approach can cause artefacts when the data falls to the border of the bins – so called bin edge effects. Moreover, standard histograms are more prone to errors when they are fitted to some functions, and particularly, when the number of observations is not very high. In the KDE approach each data point (observation of particular average pore conductance) is replaced by a bell shaped curve (usually Gaussian function) centred about the observed event value. These curves are cumulated for all events into the final curve which shows a more representative picture of variability of the observed quantity. Recently adopted variant of this approach is the violin plot. We use a very simple custom made script for generation of conductance KDE. There is no dramatic difference between different kernel shapes when creating KDE plot. Only the width of the kernel changes the resolution. Our custom made Perl script follows:

#! /usr/bin/perl -w

# usage: ./kde.pl  input_file column_index

$height=1; # weight of each kernel

$points=4000;# points of the histogram

$hwhm=0.5;   # Half-width at half-maximum of the kernel

$exponent=2; # shape of the kernel: 2=gaussian, >1000=rectangle

$a=$ARGV[0];# data file name

$c=$ARGV[1];# data column index from 0

$max=200; # max. histogram value

$min=-20; # min. histogram value

$log2=log(2);

$step=$max/$points;

$x[0]=$min;

# Load the data:

open(AA,$a);# open data file

$i=1;

while($i<$points+1){

  $sum[$i]=0;

  $x[$i]=$x[$i-1]+$step;

  $i++;

}

# Create KDE “histogram”:

$i=0;

$aai=0;#index

while ($rad=<AA>){

   chomp($rad);

   $rad=~ s/^\s*//;

   @arad=split(/[[:space:]]+/,$rad); # separator?

   $aax[$aai]=$arad[$c];

   $i=0; $sum[$i]=0;

   while($i<$points+1){        $sum[$i]=$sum[$i]+$height*exp(-$log2*(($x[$i]-$aax[$aai])/$hwhm)**$exponent);

    $i++;

   }

   $aai++;

}

close(AA);

# Print the final KDE table:

$i=0;

while($i<$points+1){

 printf  "%9.4f %9.6f\n",$x[$i],$sum[$i];

 $i++;

}
